# Correlative light-electron microscopy using small gold nanoparticles as single probes

**DOI:** 10.1038/s41377-023-01115-4

**Published:** 2023-03-29

**Authors:** Iestyn Pope, Hugh Tanner, Francesco Masia, Lukas Payne, Kenton Paul Arkill, Judith Mantell, Wolfgang Langbein, Paola Borri, Paul Verkade

**Affiliations:** 1 School of Biosciences, Cardiff University, Museum Avenue, Cardiff, CF10 3AX UK; 2 School of Biochemistry, University of Bristol, University Walk, Bristol, UK; 3 School of Physics and Astronomy, Cardiff University, The Parade, Cardiff, CF24 3AA UK; 4 Present Address: Department of Chemistry, Umeå University, Umeå, 90187 Sweden; 5 Present Address: School of Medicine, University of Nottingham, Nottingham, NG7 2RD UK

**Keywords:** Multiphoton microscopy, Biophotonics

## Abstract

Correlative light-electron microscopy (CLEM) requires the availability of robust probes which are visible both in light and electron microscopy. Here we demonstrate a CLEM approach using small gold nanoparticles as a single probe. Individual gold nanoparticles bound to the epidermal growth factor protein were located with nanometric precision background-free in human cancer cells by light microscopy using resonant four-wave mixing (FWM), and were correlatively mapped with high accuracy to the corresponding transmission electron microscopy images. We used nanoparticles of 10 nm and 5 nm radius, and show a correlation accuracy below 60 nm over an area larger than 10 µm size, without the need for additional fiducial markers. Correlation accuracy was improved to below 40 nm by reducing systematic errors, while the localisation precision is below 10 nm. Polarisation-resolved FWM correlates with nanoparticle shapes, promising for multiplexing by shape recognition in future applications. Owing to the photostability of gold nanoparticles and the applicability of FWM microscopy to living cells, FWM-CLEM opens up a powerful alternative to fluorescence-based methods.

## Abstract (web summary)

Correlative light electron microscopy using individual gold nanoparticles in human cells as single probes directly visible in light microscopy by four-wave mixing imaging with high localisation precision.

## Graphical abstract

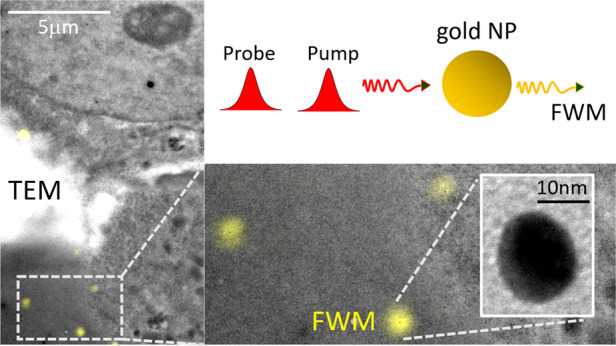

## Introduction

Correlative light-electron microscopy (CLEM) combines the strengths of light microscopy (LM) and electron microscopy (EM) and is receiving growing attention in the life sciences, especially after the recent revolutionary developments of super-resolution (SR) light microscopy and cryo-EM^1,2^. CLEM aims to combine the live cell imaging capability, large field of views, and molecular specificity of LM with the spatial resolution and ultrastructural information of EM, to pinpoint specific events and visualise molecular components in the context of the underlying intracellular structure at nanometric to atomic resolution. To highlight biomolecules of interest and determine their position with high accuracy in this context, they need to be labelled with probes that are visible both in the light microscope (typically by fluorescence) and in the electron microscope (electron-dense material). The production and detection of appropriate probes for each imaging modality is one of the key aspects of any correlative microscopy workflow.

A commonly used approach is to combine a fluorescent moiety together with a gold nanoparticle (AuNP)^3,4^. Such dual probes can be made fairly easily and are also available commercially. For example, we have used an Alexa594 fluorescent dye and a 5 nm diameter AuNP coupled to the ligand transferrin (Tf), a molecule that normally recycles between the plasma membrane and early endosomes. Importantly, we showed that such a conjugate was trafficking as expected, i.e., the function of Tf was not perturbed by the probe^3^. However, the fluorescence of Tf-Alexa594 with the AuNP was diminished compared to Tf-Alexa594. Indeed, fluorescence quenching, due to nonradiative transfer in the vicinity of an AuNP, is a well-documented effect, which can significantly reduce the applicability of these probes in CLEM workflows^5,6^. Moreover, we have shown recently that the integrity of this type of dual probe inside cells, and in turn their ability to correlatively report the location of the same molecule, should be seriously questioned^7^.

Ideally one would like to use a single probe that is visible both in the light and in the electron microscope. Semiconductor nanocrystals, also called quantum dots (QDs), do represent a single CLEM probe as they harbour an electron-dense core that also emits fluorescence^8^. However, QDs typically contain cytotoxic atoms (e.g., Cd or As). In turn, they require a protective shell coating for bio-applications which can double the probe size^8^. Moreover, QDs have an intermittent ‘on-off’ emission (i.e., they blink)^9^. This limits their application, e.g., in time-course experiments aimed at tracking the same probe over time, whereby blinking causes problems when trying to reconnect positions to generate long trajectories.

Alternately, there have been some developments toward using fluorophores as single probes^10,11^. However, this is challenging since the fixation and staining protocols for EM are often not compatible with retaining fluorescence emission. Fluorescence imaging after sample preparation for EM is key to minimising the uncertainty regarding the relative positions of fluorescent labels and EM structural features, due to the anisotropic shrinking and deformations caused by the sample processing steps. With the advent of cryo-EM which can directly image biomaterials without staining and offers the best approach to preserve the native cellular ultrastructure, workflows have been developed to perform light microscopy at cryogenic temperature^12,13^. Notably, cryo-LM has the added benefit of an increased photostability of organic fluorophores at low temperatures, which has been exploited to achieve super-resolution fluorescence microscopy with selected photo-switchable fluorescent proteins^13,14^, reducing the resolution gap between LM and EM modalities. However, cryo-LM is technically challenging, often requiring sophisticated custom setups with highly stable cryostages, and specific high numerical aperture (NA) long-working-distance air objectives to avoid sample devitrification. Moreover, the requirement for high light intensities onto the sample to achieve SR can cause sample devitrification and damage, and preclude subsequent imaging using cryo-EM. It is also important to point out that to achieve the highest correlation accuracy between LM and EM images, the addition of spherical bead fiducial markers that are visible in both modalities is typically required^10,13^. By measuring and matching the coordinates of the centroid of each fiducial marker in the LM image and the EM image, one can calculate the transformation between the two images, which takes into account changes in magnification, rotation, and distortions. However, introducing fiducials adds further steps to the sample preparation protocols, increasing complexity and possible artefacts by induced modifications.

Another approach would be to use small AuNPs as single probes. These are easily visible in EM, and exhibit strong light scattering and absorption at their localised surface plasmon resonance (LSPR). They are photostable, and the achievable photon fluxes are governed by the incident photon fluxes and the AuNP optical extinction cross-section, a significant advantage compared to fluorophores which can emit a maximum of one-photon per excited-state lifetime. However, when small AuNPs are embedded inside scattering and autofluorescing cellular environments, it is challenging to distinguish them against this background using conventional one-photon (i.e., linear) optical microscopy methods. Recently, we developed a multiphoton LM technique which exploits the four-wave mixing (FWM) nonlinearity of AuNPs, triply resonant to the LSPR. With this method, we were able to detect individual small (down to 5 nm radius) AuNPs inside scattering cells^7,15^ and tissues^16^ completely free from background, at imaging speeds and excitation powers compatible with live cell imaging, with a sensitivity limited only by photon shot noise.

Here, we demonstrate a CLEM workflow using individual small AuNPs as single probes of the epidermal growth factor (EGF) protein in mammalian cancer cells, imaged by FWM in LM and correlatively by transmission EM. Owing to the high photostability of AuNPs under ambient conditions, cryo-LM is not required in this workflow. To preserve the cellular ultrastructure and avoid artefacts from chemical fixation, we use vitrification by high-pressure freezing (HPF), followed by freeze substitution and resin embedding without additional heavy metal stains^17,18^. Importantly, sections are imaged by FWM after sample preparation for EM, and a direct correlation with high accuracy is demonstrated using the very same AuNP observed under both modalities, without the need for additional fiducial markers.

## Results

### Background-free four-wave mixing microscopy on EM-ready sections

**Fig. 1:**
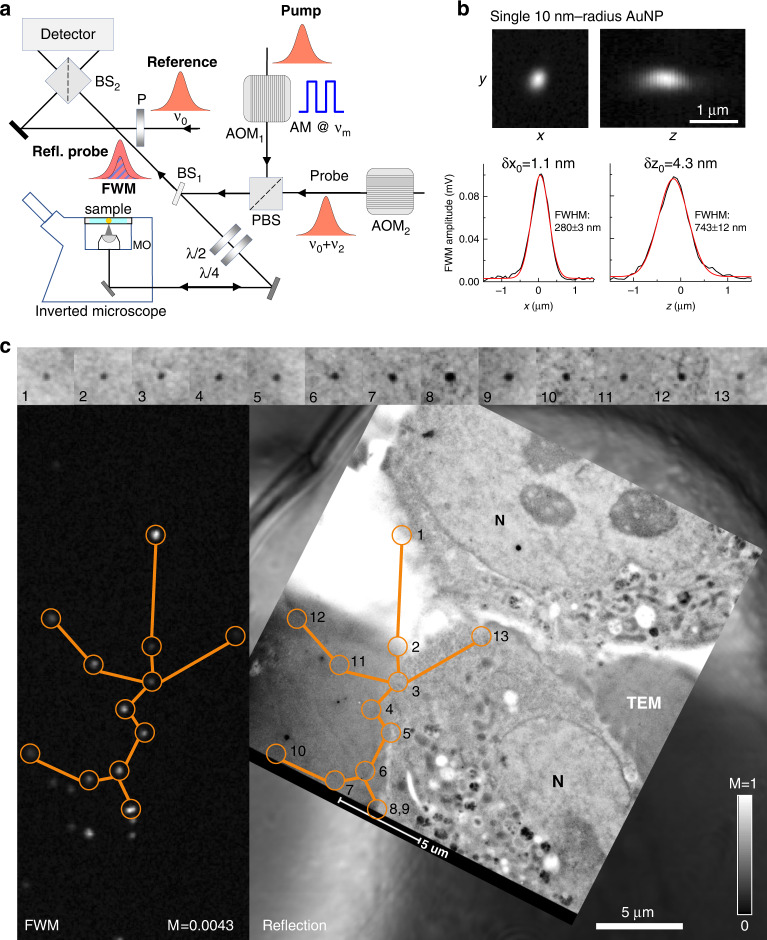
Correlative light electron microscopy using FWM imaging. **a** Sketch of FWM setup. Short optical pulses in resonance with the LSPR of AuNPs are focused onto the sample, using an inverted microscope, and generate a FWM field which is collected in epi-geometry, detected with a heterodyne interference scheme (see Methods). AOM: acousto-optic modulator. (P)BS: (polarising) beam splitter. P: polariser. MO: microscope objective. **b** Example of volumetric FWM microscopy on a single 10nm-radius AuNP, with line-profiles along *x* and *z* at the *y*-position in the centre of the AuNP and corresponding Gaussian fits (red lines). The centroid localisation precision (*δx*_0_, *δz*_0_) and the fullwidth at half maximum (FWHM) obtained from the fit are indicated. **c** CLEM of 10nm-radius AuNPs bound to the EGF protein in HeLa cells. Individual AuNPs are detected background-free in FWM (left), measured directly on 300 nm thick resin sections post-cell fixation, ready for EM analysis. The same pattern is found in TEM, highlighted by the orange circles. Two cells are visible, with their nucleus indicated (N). The nucleus is surrounded by the organelle-containing cytoplasm. The top row shows crops (0.2 µm × 0.2 µm) of the TEM image for each AuNP as numbered. The confocal reflection image simultaneously acquired with FWM is shown underneath the TEM image. Greyscales are from 0 to M as indicated (M = 1 correspond to 31 mV rms detected, see also Methods for details of the excitation and detection conditions)

In its general form, FWM is a third-order nonlinear light-matter interaction phenomenon wherein three light fields interact in a medium to generate a fourth wave. Here, we use a scheme where all waves have the same centre frequency, and two of the incident light fields are identical (two-beam degenerate FWM). A sketch of the experimental setup implementing the FWM technique is shown in Fig. 1a. It exploits a combination of short optical pulses of about 150 fs duration, called pump, probe and reference, generated by the same laser source (see also Methods). All pulses have the same centre optical frequency, in resonance with the localised surface plasmon of nominally spherical small AuNPs. The detected FWM can be understood as a pump-induced change in the AuNP dielectric function, which manifests as a change in the scattering of the probe beam^15^. Pump and probe pulses are focused onto the sample using a high NA microscope objective (MO), and the FWM signal is collected by the same objective in reflection (epi-geometry). To distinguish FWM from pump and probe beams, a heterodyne detection scheme is implemented, wherein the pump is amplitude modulated (at *ν*_m_), the probe is radiofrequency shifted (by *ν*_2_), and the interference between FWM and reference fields is detected at the appropriate radiofrequency sidebands *ν*_2_ ± *ν*_m_ (modulo the laser repetition rate^15^). We have shown previously that the maximum FWM field amplitude is detected when the probe pulse arrives about 0.5 ps after the pump pulse, which corresponds to the time needed for the free electron gas in the metal to reach the highest temperature (due the transfer of energy from the pump absorption) before starting to cool down via electron-phonon scattering^19^. As a result of this detection scheme, FWM is free from both linear scattering and incoherent (e.g., autofluorescence) background, and is temporally separated from instantaneous as well as long-lived non-linearities. Such exquisite background-free contrast is showcased in Fig. 1c, where FWM was acquired on AuNPs of nominal 10 nm radius bound to the EGF protein in HeLa cells, measured on 300 nm thin sections ready for EM analysis, prepared using cell fixation by HPF followed by freeze substitution and resin embedding (see Methods). Although these samples are embedded in Lowicryl HM20 resin without the addition of any electron-dense staining agents, the sections create a strong background in the linear response, as shown in the confocal reflectance image acquired simultaneously with FWM in Fig. 1c. Yet, FWM is free from background and clearly shows the location of individual AuNPs (highlighted by the orange circles in Fig. 1c). The identical AuNP spatial pattern is found in the transmission EM (TEM) of the same section, correlatively measured after FWM imaging (see Methods), showcasing the suitability of AuNPs as single probes visible with high contrast in both FWM and EM. Notably, it is possible to locate the centroid position of single AuNPs in an FWM image with a localisation precision much better than the diffraction-limited spatial resolution, as shown in Fig. 1b. Gaussian fits of one-dimensional line profiles along *x* and *z* at the *y*-position in the centre of a single AuNP provide a centroid localisation precision of about 1 nm in-plane and 4 nm axially for the signal-to-noise ratio in the data. Furthermore, the FWM field phase in reflection encodes the axial displacement between the particle and the focus centre, thus it can be used to determine the particle *z* coordinate without axial scanning^15^. The linear dependence of the FWM phase versus *z* measured on a set of AuNPs is reported in Supplementary Information (SI) Fig. S1.

### FWM is sensitive to the AuNP shape

It was shown in our previous work^15^ that using a polarisation-resolved configuration in the FWM field detection provides additional information on the AuNP shape and orientation. In this configuration, probe and pump beams, linearly polarised in the laboratory system, are transformed into circularly polarised beams at the sample by a combination of *λ/*4 and *λ/*2 waveplates (see also Fig. 1a).

We then use a dual-polarisation balanced detection (see Methods) which allows us to detect the co- and cross-circularly polarised components of the reflected probe and FWM fields relative to the incident circularly polarised probe, having amplitudes (phases) indicated as $A_{{{{\mathrm{2r}}}}}^ \pm$ and $A_{{{{\mathrm{FWM}}}}}^ \pm$ $\left( {{{{\mathrm{{\Phi}}}}}_{{{{\mathrm{2r}}}}}^ \pm \,{{{\mathrm{and}}}}\,{{{\mathrm{{\Phi}}}}}_{{{{\mathrm{FWM}}}}}^ \pm } \right)$, respectively, where +(−) refers to the co (cross) polarised component. Notably, we found, with the aid of numerical simulations of the detected FWM field spatial pattern compared with the experiments, that the cross-polarised component is strongly sensitive to small AuNP shape asymmetries, which are always present in these nominally spherical AuNPs consistent with their morphology observed in TEM. Using an ellipsoid model to account for deviations from spherical shapes, the calculations showed that the amplitude ratio $A_{{{{\mathrm{FWM}}}}}^ - /A_{{{{\mathrm{FWM}}}}}^ +$at the AuNP centre is proportional to the AuNP ellipticity and that the phase difference ${{{\mathrm{{\Phi}}}}}_{{{{\mathrm{FWM}}}}}^ - - {{{\mathrm{{\Phi}}}}}_{{{{\mathrm{FWM}}}}}^ +$ reports the in-plane particle orientation^15^.

**Fig. 2:**
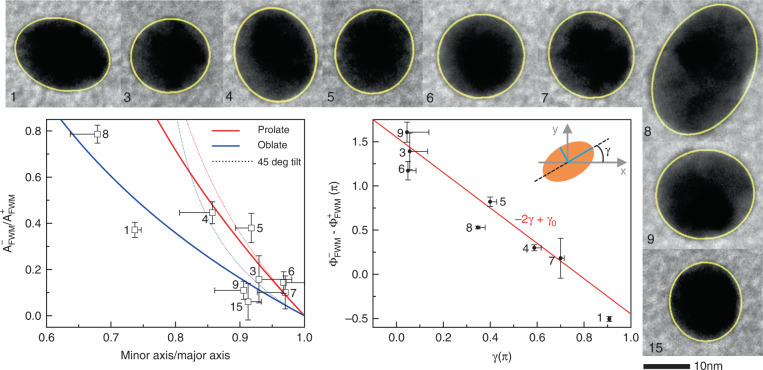
FWM dependence on the AuNP aspect ratio and orientation. High-magnification TEM images of selected nanoparticles (numbered as in Fig. 1c) were fitted with an elliptical shape (shown in yellow). AuNP 15 is not captured by the TEM overview in Fig. 1c and is shown in SI Fig. S2 and Fig. S7. The corresponding major and minor axis and the orientation angle *γ* were obtained (see also sketch). The ratio of the cross- to co-circularly polarised FWM components in amplitude ($A_{{{{\mathrm{FWM}}}}}^ - /A_{{{{\mathrm{FWM}}}}}^ +$) and phase $\left( {{{{\mathrm{{\Phi}}}}}_{{{{\mathrm{FWM}}}}}^ - - {{{\mathrm{{\Phi}}}}}_{{{{\mathrm{FWM}}}}}^ + } \right)$ is shown versus the in-plane elliptical aspect ratio and orientation angle. Vertical error bars represent the single-pixel shot noise in the FWM measurements. Single-sided horizontal bars were obtained by fitting the TEM images with a variable contrast threshold (see text). For $A_{{{{\mathrm{FWM}}}}}^ - /A_{{{{\mathrm{FWM}}}}}^ +$, red (blue) lines are calculated dependences assuming a prolate (oblate) ellipsoid with axis *a* > *b* = *c* (*a* < *b* = *c*) aligned along the *x, y, z* directions respectively. Dotted lines assume that the NP *a* and *c* axis are tilted by 45 degrees in the *x, z* plane, having a projected axis in the *x* direction as derived in Section S3.i

Using the CLEM workflow, here we have correlatively analysed the measured FWM field ratio and the AuNP shape obtained with TEM, and compared the results with the ellipsoid model previously developed. Fig. 2 shows high-magnification TEM images on a selection of the AuNPs seen in Fig. 1c, as indicated by the corresponding numbers. An ellipse was fitted to these images as shown by the yellow lines (see also Methods). The corresponding major and minor axis and the orientation angle *γ* were obtained (see the sketch in Fig. 2) and the dependence of the measured FWM field ratio at the AuNP centre is shown in the plots, for both amplitude and phase components. Error bars in the measured FWM field ratio represent the shot-noise in regions away from the AuNPs while the horizontal error bars were obtained by changing the threshold levels used to fit an ellipse to the TEM images (see Methods). For this analysis, we ensured that the selected NPs were sufficiently in focus (see SI Fig. S4), to justify comparing the experimentally measured FWM ratio with the ellipsoid model. The latter was developed assuming a prolate or an oblate NP shape, with semiaxis *a* > *b* = *c* or *a* < *b* = *c* along the *x, y, z* directions, respectively. We also considered the case of a tilted ellipsoid rotated by 45 degrees in the *x, z* plane, and calculated the projected semiaxis along *x* accordingly (see SI section S3). The corresponding amplitude ratios $A_{{{{\mathrm{FWM}}}}}^ - /A_{{{{\mathrm{FWM}}}}}^ +$derived from such model are shown in Fig. 2 as labelled. Generally, the experimental data agree well with the model, taking into account that the TEM used here is an in-plane projection of the 3D shape, hence we cannot tell if a NP is oblate or prolate and how its axes are orientated. Notably, NPs number 1 and 15 show a darker contrast in TEM, consistent with having an oblate shape with the long *c* axis out of plane. Regarding the NP in-plane orientation, the experimental FWM ratio phase ${{{\mathrm{{\Phi}}}}}_{{{{\mathrm{FWM}}}}}^ - - {{{\mathrm{{\Phi}}}}}_{{{{\mathrm{FWM}}}}}^ +$ exhibits a good agreement with the dependence −2*γ* + *γ*_0_, where *γ*_0_ is a rotation offset, as predicted by the ellipsoid model^15^.

### FWM-EM correlation accuracy

The correlation accuracy between locating the same AuNP in FWM and in TEM was evaluated as follows. The centroid **r**_**0**_ = (*x*_0_*,y*_0_) position coordinates of each individual AuNP in a FWM image were obtained using a two-dimensional Gaussian fit of the $A_{{{{\mathrm{FWM}}}}}^ +$ profile (see Methods). The corresponding AuNP coordinates in the EM were assigned by examining zooms at the particle location and positioning the particle centre based on shape geometry. The two sets of coordinates were then compared using a linear transformation matrix. Specifically, the coordinates of each AuNP in the FWM image were transformed into the reference system of the EM using an affine transformation **C**, including shear, scaling, rotation and translation, so that **r**_**B**_ = **C**(**r**_**A**_) where **r**_**A**_ is the coordinate vector in the FWM image and **r**_**B**_ is the coordinate vector in the EM image. For more than 3 AuNPs, the system is overdetermined and **C** is calculated by minimising the sum of the squared deviations over all particle coordinates (see also Methods). As a measure of the correlation accuracy we then evaluate the quantity $S = \sqrt {\frac{1}{N}\mathop {\sum}\nolimits_i {\left| {r_{B_i} - C\left( {r_{A_i}} \right)} \right|} ^2}$ where *N* is the total number of AuNPs being compared and *i* = 1*,….,N* denotes the individual i-th particle.

**Fig. 3:**
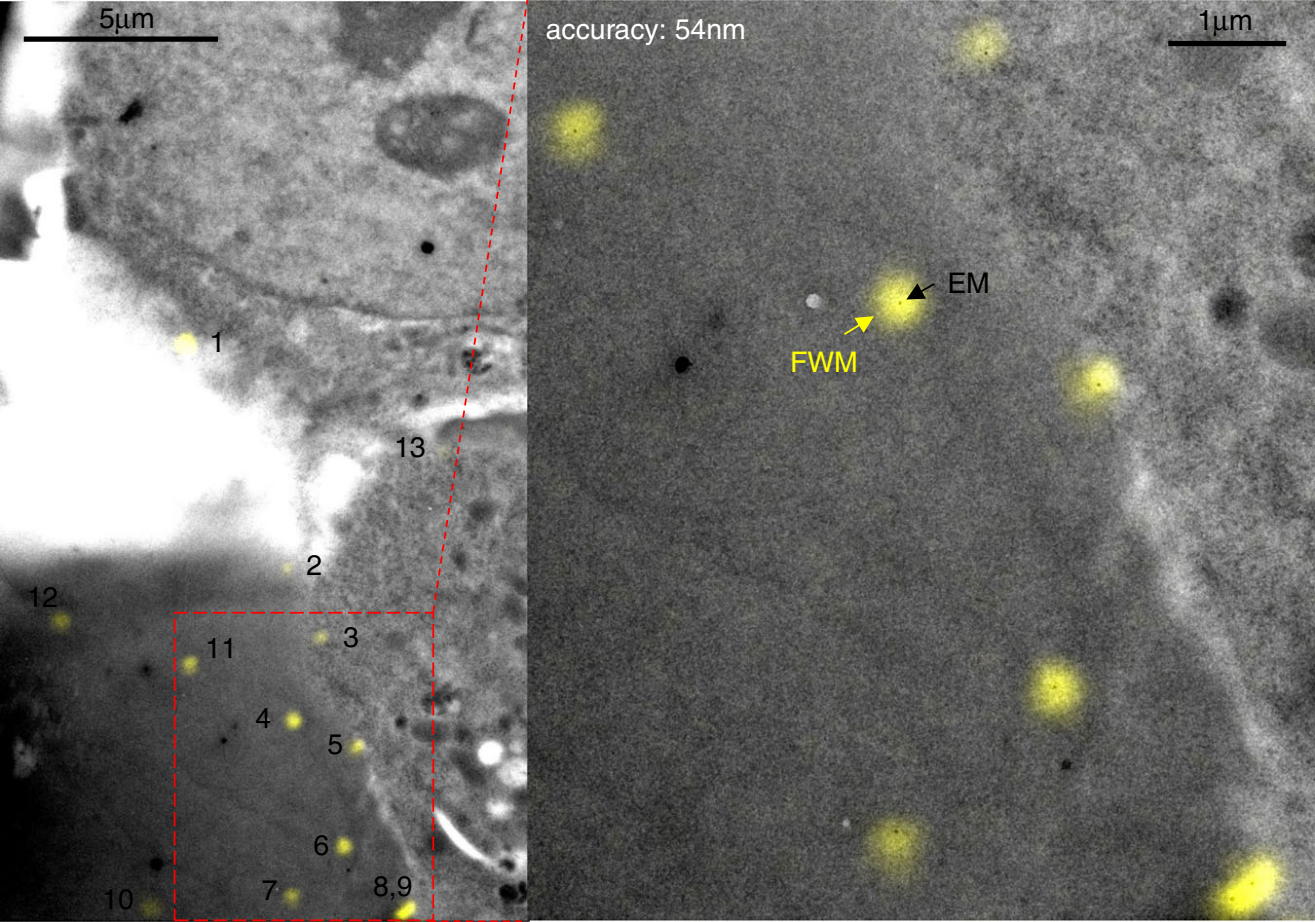
CLEM correlation accuracy. Overlay of FWM field amplitude (yellow) and TEM image (grey) from Fig. 1c (contrast adjusted for visibility). The FWM image is transformed into the EM reference system using a linear transformation matrix that accounts for translation, rotation, shear and scaling (see text). The correlation accuracy is indicated

**Fig. 4:**
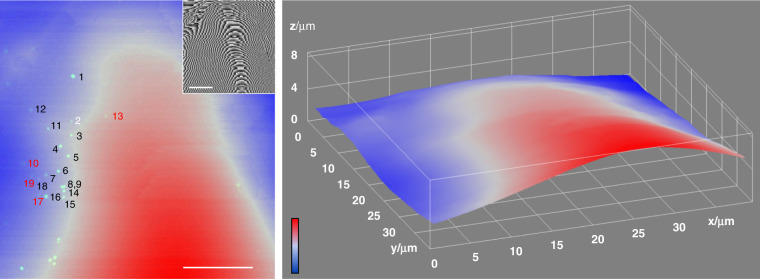
Surface height profile of resin section in FWM imaging. Colour-coded height profile (blue: 0, red: 8.51 µm) obtained from the phase of the reflected probe field, shown in the inset on a grey scale from −*π* (black) to *π* (white), for the region in Fig. 1c and Fig. S7. The FWM field amplitude of AuNPs is overlaid on the left panel and AuNPs are labelled. The surface profile shows height differences of several microns, indicating a ripple in the pioloform layer and the supported 300 nm thin resin section. AuNPs labelled in red are those excluded from the CLEM correlation analysis as they are too out of focus (AuNP 10 is 1.9 µm below and AuNP 13 is 0.8 µm above AuNP 5). Scale bar: 10 µm

Fig. 3 shows an example of this analysis for the 10nm-radius AuNPs reported in Fig. 1c, where the transformed FWM image has been overlaid with the EM image. A zoom of the overlay is given in Fig. 3 to showcase the overlap between an individual AuNP in FWM (yellow spots) and EM (black spots). A correlation accuracy of 94 nm is found when including all numbered particles in Fig. 1c, which reduces to 54 nm when excluding AuNPs 2, 10, and 13. These three particles have a FWM amplitude below a third of the typical maximum value observed. A high-magnification TEM inspection of AuNP 2 shows a weak contrast (see SI Fig. S5), hence an atypical structure, while AuNP 10 and 13 have a low FWM amplitude because they are significantly out of focus, as demonstrated by an analysis of the point-spread-function (PSF) width and the error in the centroid localisation precision (see SI Section S4, Fig. S6). When particles are out of focus, not only the localisation precision decreases but their location is also affected by additional uncertainties, including objective aberrations and deformations of the pioloform layer supporting the resin section which change from FWM in water to EM in vacuum (see Methods). Notably, by exploiting the topography information encoded in the detected phase of the reflected probe field, we reconstructed a height profile of the resin section for the region in Fig. 1c, showing that there is a vertical tilt/bending of the pioloform layer, and AuNP 10 and 13 are indeed located at significantly different heights compared to the other particles (see SI Section S4, and Fig. 4 which shows AuNP 10 being 1.9 µm below and AuNP 13 being 0.8 µm above AuNP 5). This also explains why, despite the resin section being only 300 nm thick, hence smaller the axial extension of the PSF in FWM imaging (as shown in Fig. 1b), we do have issues of AuNPs being out of focus.

It should be highlighted that a correlation accuracy of 54 nm is remarkably small considering the large size (*>*10 µm) of the region over which the correlation is carried out. An additional example using a different, slightly smaller, EM region (centred around AuNPs 8 and 9) is shown in Fig. S7, giving a correlation accuracy of 43 nm, when excluding AuNP 17 and 19 from the analysis after consistently applying the same out-of-focus criteria mentioned above (see SI Section S4, Fig. S6 for details).

**Fig. 5:**
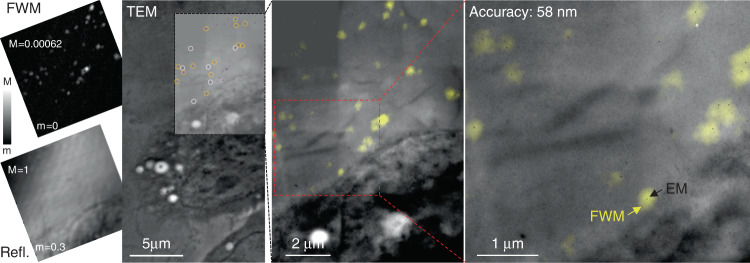
CLEM correlation accuracy with 5nm-radius AuNPs. HeLa cells incubated with 5nm-radius AuNPs bound to the EGF protein. Individual AuNPs are detected background-free in FWM (left) measured on 300 nm thin resin sections post-cell fixation, ready for EM analysis. The confocal reflection image simultaneously acquired with FWM is shown below (linear grey scales are from m to M as indicated; M = 1 correspond to 65 mV rms detected, see Methods for details of the excitation and detection conditions). A large overview TEM of the same region is shown. On the area indicated by the black dashed frame, a series of high resolution EM images were taken and stitched together. Individual AuNPs are highlighted by the circles. The overlay between FWM (yellow) and TEM (grey) is shown on the centre and further zoomed into the indicated red dashed area on the right side (contrast adjusted to aid visualisation). For the correlation analysis, of the 19 individual AuNPs highlighted by the circles, 6 (white circles) were discarded as being of focus. The FWM image was transformed into the EM reference system using a linear transformation matrix that accounts for translation, rotation, shear and scaling of axes. On the right side, individual AuNPs identified in FWM (yellow spots) are seen in EM (black dots). The correlation accuracy is indicated

We also investigated HeLa cells incubated with 5 nm-radius AuNPs. It was shown in our previous work^19^ that the FWM field amplitude scales almost proportionally with the AuNP volume. Therefore, the signal-to-noise ratio, and in turn the localisation precision, is decreased by approximately eightfold compared to using 10 nm-radius AuNPs under identical excitation and detection conditions. Still, individual nanoparticles of this small size can be clearly resolved in FWM microscopy, above noise and background-free, as we showed in ref. ^7^. An example of CLEM with FWM imaging using 5 nm-radius AuNPs in HeLa cells is shown in Fig. 5. Several AuNPs are clearly visible in both FWM and TEM. A few AuNPs are too close to be spatially distinguished in the FWM image, but 19 individual AuNPs are available for position analysis. This resulted in a correlation accuracy of 58 nm, whereby 13 individual AuNPs were used for the correlation (see orange circles in Fig. 5), and 6 nanoparticles were excluded (white circles in Fig. 5) based on the out-of-focus criteria discussed previously (see SI Section S4, Fig. S8). Another example showing an adjacent region is provided in Fig. S9. Merging both regions results in a correlation accuracy of 63 nm (see SI section S4).

We should note that the value *S* scales with the number of particles included in the analysis *N* and the number *M* of parameters in the transformation according to $\sqrt {\left( {2N - M} \right)/\left( {2N} \right)}$. In other words, decreasing the number of particles in the analysis decreases the quantity *S* (as stated above, if *N* = 3 the M = 6 parameters of **C** are fully determined from linear algebra and *S* = 0). To account for this, we can calculate a corrected correlation accuracy as

$S/\sqrt {1 - \left( {M/2N} \right)}$. This is found to be 65 nm both for the 10nm-radius AuNPs in Fig. 3 and for the 5nm-radius AuNPs in Fig. 5. We also note that our analysis considers all particles as useful localisable objects of interest, visible in both FWM and EM, with no need for additional fiducial markers. In the SI Section S4 we discuss an alternative analysis which considers three particles as fiducials to determine the transformation parameters of **C**, and calculates the overlay accuracy of the remaining particles.

**Fig. 6:**
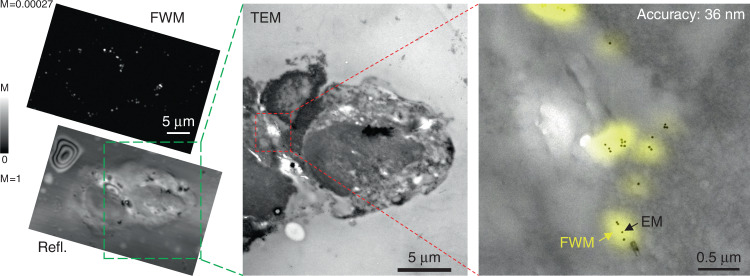
CLEM correlation accuracy with 3D FWM analysis. FWM-CLEM using 10 nm-radius AuNPs bound to EGF internalised in HeLa cells whereby the coordinates of the particles in FWM are measured in 3D via a z-stack. A large FWM overview in 2D with corresponding reflection image measured simultaneously is shown on the left (linear grey scales are from 0 to M as indicated; M = 1 corresponds to 33 mV rms detected; see Methods for details of the excitation and detection conditions). A TEM overview of the same region is shown in the centre, as indicated by the green dashed frame. On the right, an overlay of FWM field amplitude (yellow) and TEM image (grey) is shown for the region highlighted by the red dashed frame, where FWM is a maximum amplitude projection from a 3D z-stack (50 nm step size in *z*). AuNPs form small clusters and are no longer resolved as individual particles in FWM. The centroid position of each cluster was determined in 3D from the FWM z-stack (see Methods), and its 2D in-plane coordinates were compared with the position of the geometrical centre of the cluster in TEM (which is a 2D transmission projection) for the correlation analysis. The resulting correlation accuracy from the comparison of the six clusters shown in the figure is indicated

Considering that the shot-noise-limited precision in locating the centroid position of a AuNP in focus by FWM is only a few nanometres (see Fig. 1b), the measured values of *S*, even after excluding AuNPs which are too out of focus, are limited by systematic errors, i.e., *S* is dominated by accuracy rather than precision. To address this point, we performed FWM-CLEM using 10nm-radius AuNPs whereby the coordinates of the particles in FWM were measured in 3D with a fine axial scan (50 nm step size in *z*), such that the coordinates at the plane of optimum focus are accurately determined and systematics from e.g., out-of-focus aberrations are eliminated. These results are summarised in Fig. 6. Notably, here we observe AuNPs which have been internalised inside the cells (instead of being outside or at the cell surface, as in Fig. 1c). AuNPs form small clusters and are no longer resolved as individual particles in FWM. Therefore, in this case, we determined the centroid position of the cluster in 3D from the FWM z-stack (see Methods), and compared its 2D in-plane coordinates with the position of the geometrical centre of the cluster in TEM (which is a 2D transmission projection) for the correlation analysis. The resulting correlation accuracy for the six clusters shown in Fig. 6 is 36 nm. Another example correlating 10 clusters is provided in Fig. S10, for which an accuracy of 44 nm is found.

## Discussion

The demonstration of FWM-CLEM with a single AuNP probe opens new possibilities for CLEM workflows. As shown here, we can locate the position of a single AuNP with nanometric precision at ambient conditions, without the need for cryo light microscopy, owing to the background-free and photostable FWM response of individual AuNPs which do not photobleach. The very same AuNP is well visible in EM due to its electron-dense composition, offering high correlation accuracy without the need for additional fiducials. We have shown proof-of-principle results with 10 nm-radius and 5 nm-radius AuNPs bound to the EGF protein in HeLa cells, using FWM directly on 300 nm thin sections prepared for EM by HPF, freeze substitution and Lowicryl HM20 resin embedding without using heavy metal stains.

Generally, we found a correlation accuracy limited by systematics, in the range of 60 nm or less over areas larger than 10 µm. Systematic errors included a bending of the pioloform layer supporting the resin section, which changes from FWM in water to TEM in vacuum. This is difficult to correct for by coordinate transformations, and likely to require non-trivial methods beyond the linear transformation used by us. Importantly, systematics can be improved in future experimental designs, such that a correlation uncertainty eventually limited only by localisation precision from photon shot-noise, and hence down to 5–10 nm (or even lower by measuring longer, considering the photostability of AuNPs) could be reached. Moreover, since a single probe is used, as soon as this is identified from FWM into the TEM image, its relationship with the cellular ultrastructure is unambiguously determined. We should also highlight that FWM is compatible with live cell imaging^16^, hence can be applied from the start of a CLEM workflow, before cell fixation, as well as post fixation.

The detection of individual AuNPs with FWM lends itself to applications in single particle tracking (SPT) inside living cells^20^, to follow e.g., the entry and intracellular pathways of single molecules tagged with AuNPs, from proteins to drugs. A related application is following the fate of individual virions^21^ to gain spatio-temporal insights into fundamental mechanisms of virus transport and infection occurring in live cells. Combined with existing strategies to label with or even encapsulate AuNPs inside virions^22^, FWM opens the exciting prospect to track single virions over long observation times, background-free and deep inside living cells and tissues, to then pin-point events of interest (e.g., genome release) in the context of the cellular ultrastructure by CLEM.

While in the present demonstration we have shown AuNPs probes down to 5 nm radius, we emphasise that smaller probes could be used. In fact, in our previous work^23^ we reported FWM microscopy with 2.5 nm radius AuNPs immunostaining the Golgi apparatus of HepG2 cells, where nanoparticles were detected as clusters in the focal volume. The FWM field amplitude scales proportionally with the NP volume and with the number of isolated particles in the focal volume, thus eight AuNPs of 2.5 nm-radius provide the same FWM signal as a single 5 nm-radius AuNPs under the same excitation and detection conditions. The FWM amplitude signal-to-noise ratio scales as $\sqrt t I_1\sqrt {I_2}$ with *I*_1_ (*I*_2_) being the intensity of the pump (probe) beam at the sample and *t* the integration time^15^, hence to detect a single 2.5 nm radius AuNP (instead of a cluster) one can increase the excitation power and integration time accordingly. However, these conditions might prevent the applicability of the technique to living cells, due to nanoparticle heating under high power illumination and/or integration times becoming too long for the dynamics under observation. Alternatively, 2.5 nm radius silver nanoparticles can be used, as these have a 10-fold larger polarisability compared to a AuNP of equal radius (and correspondingly will exhibit higher FWM), owing to their sharper LSPR in the absence of interband transitions, as was seen in their photothermal response^24^. Gold nanorods also have sharper LSPR due to the redshift of the longitudinal plasmon resonance away from interband transitions^25^. Calculations of the optical absorption cross-section^26^ indeed show that a gold nanorod with 2.5 nm radius and 5 nm long semiaxis has an absorption cross-section, for the longitudinal LSPR, 10-fold larger compared to a spherical AuNP of 2.5 nm radius, hence correspondingly stronger FWM.

It should be mentioned that a limitation of the present FWM implementation is the imaging speed of ~0.1 Hz frame rates for a typical image of 100 × 100 pixels at 1 ms pixel dwell time. Shorter acquisition times can be used (albeit reducing signal-to-noise ratio proportional to $\sqrt t$ as mentioned above) and we have shown ~0.1 ms pixel dwell times with AuNPs of 10 nm radius or larger^7,15^. Eventually, however, our imaging speed is limited by the slow response time (~10 ms) of the piezoelectric stage used to move the sample (see Methods for details of the hardware). This limitation can be overcome by beam scanning with fast galvo mirrors. Another way forward could be to implement a wide-field FWM illumination and camera detection, removing the need for beam/sample scanning, potentially reaching kHz frame rates with presently available high-speed cameras. Notably, FWM imaging requires low excitation intensities (*<*1 mW/µm^2^), reachable on a wide-field illumination area of ~1000 µm^2^ using average laser powers of ~1 W which are available from existing pulsed laser sources.

Let us also discuss the opportunities and drawbacks of FWM imaging within the range of EM sample preparation protocols typically used. Here, we have shown cells fixed by HPF, freeze substitution and resin embedding without heavy metal stains. These metal stains are often introduced in EM to enhance the visibility of the cellular ultrastructure. However, standard stains such as Osmium and Uranyl acetate also generate a significant photothermal response (namely a light-induced heating of the material which changes its refractive index and modulates the probe reflection) affecting the FWM imaging contrast. Without metal stains, the EM contrast in the ultrastructural definition of cell organelles is indeed low, although this can be improved using electron tomography and applying an average intensity z-projection onto a subset of reconstructed slices (see SI Fig. S11). Interestingly, Lanthanide acetates have been shown to provide a viable alternative to standard metal stains in EM, particularly to replace Uranyl acetate since uranium compounds are nuclear fuel materials and are tightly controlled worldwide^27,28^. Lanthanides such as Sm and Eu as 3+ ions in solution have only a weak optical absorption in the visible wavelength range, due to their electronic configuration with the 4 f shell as the highest occupied^29^. Therefore, we expect their photothermal response in FWM to be negligible. The use of lanthanide stains is thus a promising avenue to improve the ultrastructural definition of cell organelles in FWM-CLEM without the drawback of reducing FWM contrast.

Notably, metal stains are avoided in cryo-EM, to maintain sample integrity and image intact protein structures at high resolution. On that note, albeit technically challenging, it would be interesting to explore FWM in the context of a fully cryo-CLEM workflow. A key question for this implementation is the extent to which the illumination powers and intensities required in FWM imaging would cause specimen heating and devitrification. As a single point scanning technique, excitation *powers* are low (10–100 µW) in FWM, as compared to wide-field fluorescence single molecule localisation microscopy methods using 10–100 mW total powers and requiring high thermal conductivity substrates to minimise sample heating and maintain cryo conditions^13,14^. FWM excitation intensities are comparable to or lower than those used in confocal fluorescence microscopy, but could be too high for cryo-LM, especially considering the local heating of the AuNP occurring in FWM. Albeit transiently (~10 ps) the AuNP temperature can be very high, we calculated that the time-average AuNP temperature increase is below 10 degrees in typical FWM measurements^19^. Notably, although these calculations were for experiments at room temperature, due to the low Debye temperature of gold, the heat capacity is decreasing by only 20% at 77 K^30^, thus not significantly altering the estimated AuNP temperature increase. For biological cryo samples at 77 K, such temperature change should be below the devitrification conditions^31^, suggesting that cryo-FWM could be feasible.

Another interesting consideration is the sensitivity of polarisation-resolved FWM to the shape and orientation of individual AuNPs, as we have shown here and previously^15^. From a single particle tracking standpoint, this opens the exciting prospect of tracking particle rotations as well as translations, while for imaging it provides an opportunity for multiplexing by size and shape recognition. To that end, the use of gold nanorods having a strong FWM response and a well-defined shape asymmetry is especially promising. For example, we calculated^15^ that gold ellipsoid generate a significant cross-polarised FWM field component, independent of the particle position within the focal volume even for small ellipticities (≥20%), which can be exploited as a robust reporter to track the nanoparticle rotations.

Beyond imaging, we highlight the potential of harnessing the FWM nonlinearity for sensing the local environment surrounding a AuNP. Indeed, we showed previously that the transient FWM dynamics as a function of the delay time between pump and probe pulses depend on the local thermal conductivity of the surrounding^19^. These dynamics could thus be harnessed in a way similar to fluorescence lifetime imaging microscopy, generating spatial maps of the FWM characteristic decay times which encode changes in the local thermal conductivity. Moreover, in the context of SPT, the roto-translational motion of a small AuNP can be used as a reporter of the local viscoelastic properties of the soft-matter environment in which the particle moves, accessing nanomechanical behaviours at a scale much smaller than typical micro-rheology studies^32^. Finally, we highlight the recent demonstration that AuNPs can be synthesised directly inside cells and attached to specific biomolecules using genetic tagging^33^. This could bring a “bioimaging revolution” to FWM microscopy and FWM-CLEM, similar to the advent of fluorescent proteins in fluorescence microscopy.

## Methods

### FWM setup

FWM microscopy was performed using a home-built setup, as described in detail in our recent works^7,15^. Briefly, optical pulses of 150 fs duration centred at 550 nm wavelength with *ν*_L_ = 80 MHz repetition rate were provided by the signal output of an optical parametric oscillator (Spectra Physics Inspire HF 100) pumped by a frequency-doubled femtosecond Ti:Sa laser (Spectra Physics Mai Tai HP). The output was split into three beams having the same centre optical frequency, resulting in a triply degenerate FWM scheme. One beam acts as a pump and excites the AuNP at the LSPR, with an intensity that is modulated at *ν*_m_ = 0.4 MHz by an acousto-optic modulator (AOM). The change in the AuNP optical properties induced by this excitation is resonantly probed by a second pulse at an adjustable delay time after the pump pulse. Pump and probe pulses are recombined into the same spatial mode and focused onto the sample by a ×60 water-immersion objective of 1.27NA (Nikon CFI Plan Apochromat lambda series MRD70650) mounted onto a commercial inverted microscope (Nikon Ti-U) with a 1.5× tube lens. The sample is positioned and moved with respect to the focal volume of the objective by scanning a *xyz* sample stage with nanometric position precision (MadCityLabs NanoLP200). A FWM field (proportional to the pump-induced change of the probe reflected field) is collected by the same objective (epi-detection), together with the probe reflected field, transmitted by an 80:20 (T:R) beam splitter (BS_1_ in Fig. 1) used to couple the incident beams into the microscope, and recombined in a 50:50 beam splitter (BS_2_) with a reference pulse field of adjustable delay. The resulting interference is detected by two pairs of balanced Si photodiodes (Hamamatsu S5973-02). A heterodyne scheme discriminates the FWM field from pump and probe pulses and detects the amplitude and phase of the field. In this scheme, the probe optical frequency is upshifted by a radio-frequency amount (*ν*_2_ = 82 MHz), and the interference of the FWM with the unshifted reference field is detected. As a result of the amplitude modulation of the pump at *ν*_m_ and the frequency shift of the probe by *ν*_2_, this interference gives rise to a beat note at *ν*_2_, with two sidebands at *ν*_2_ ± *ν*_m_, and replica separated by the repetition rate *ν*_L_ of the pulse train frequency comb. A multichannel lock-in amplifier (Zurich Instruments HF2LI) enables the simultaneous detection of the carrier at *ν*_2_ − *ν*_L_ = 2 MHz and the sidebands at *ν*_2_ ± *ν*_m_ − *ν*_L_ = 2 ± 0.4 MHz. As described in our previous work^15^ the setup also features a dual-polarisation scheme. Briefly, in this scheme, probe and pump beams, linearly polarised horizontally (H) and vertically (V) respectively in the laboratory system, are transformed into cross-circularly polarised beams at the sample by a combination of *λ/*4 and *λ/*2 waveplates (see Fig. 1a). The reflected probe and FWM fields collected by the microscope objective travel backwards through the same waveplates, such that the probe reflected by a planar surface returns V polarised in the laboratory system. The reference beam is polarised at 45 degree (using a polariser) prior to recombining with the epi-detected signal via the non-polarising beam splitter BS_2_. A Wollaston prism vertically separates H and V polarisations for each arm of the interferometer after BS_2_. Two pairs of balanced photodiodes then provide polarisation-resolved detection, the bottom (top) pair detecting the current difference (for common-mode noise rejection) of the V (H) polarised interferometer arms. The measured interference corresponds to the co- and cross-circularly polarised components of the reflected probe and FWM fields relative to the incident circularly polarised probe, having amplitudes (phases) indicated as $A_{{{{\mathrm{2r}}}}}^ \pm$ and $A_{{{{\mathrm{FWM}}}}}^ \pm$ $\left( {{{{\mathrm{{\Phi}}}}}_{{{{\mathrm{2r}}}}}^ \pm \,{{{\mathrm{and}}}}\,{{{\mathrm{{\Phi}}}}}_{{{{\mathrm{FWM}}}}}^ \pm } \right)$, respectively, where + (−) refers to the co (cross) polarised component.

The results in Fig. 1b, c refer to the co-polarised component and the acquisition parameters were as follows: pump-probe delay time of 0.5 ps, b) pump (probe) power at the sample of 100 µW (50 µW), 3ms-pixel dwell time, pixel size in plane of 21 nm and z stacks over 3 µm in 75 nm z steps; c) pump (probe) power at the sample of 80 µW (40 µW), 1ms-pixel dwell time, pixel size in plane of 72 nm. The FWM is shown as a maximum amplitude projection for two *xy* planes 0.5 µm separated in *z*.

The results in Fig. 5 refer to the co-polarised component and the acquisition parameters were as follows: pump-probe delay time of 0.5 ps, pump (probe) power at the sample of 100 µW (50 µW), 3ms-pixel dwell time, pixel size in plane: 43 nm.

The results in Fig. 6 refer to the co-polarised component and the acquisition parameters were as follows. 2D overview: pump-probe delay time of 0.5 ps, pump (probe) power at the sample of 20 µW (10 µW), 1 ms pixel dwell time, pixel size in plane 72 nm. 3D stack: pump-probe delay time of 0.5 ps, pump (probe) power at the sample of 20 µW (10 µW), 1 ms pixel dwell time, pixel size in plane 80 nm, 50 nm step size in *z* and 61 z steps (3 µm total range).

### Sample preparation

HeLa cells were grown on 1.5 mm wide sapphire discs (Leica Microsystems)^17^. Following a 2-hour serum starvation, EGF coupled to 5 or 10 nm radius AuNP was allowed to internalise into the HeLa cells for 20 min^3^. After a brief rinse in 20% BSA in growth medium, the disc was placed in a 0.1 mm deep membrane carrier and high-pressure frozen (EMPACT2 + RTS, Leica Microsystems)^17^. The frozen carrier was transferred under liquid nitrogen to an automated freeze substitution device (AFS2 + FSP, Leica Microsystems). Freeze substitution to Lowicryl HM20 was performed as described in ref. ^18^ with the exception that any heavy metal stain was omitted. Following UV polymerisation of the resin, 300 nm resin sections were cut and mounted onto copper slot grids on a layer of pioloform. For FWM imaging, the copper grids were mounted in water between a glass coverslip (Menzel Gläser, 24 mm × 24 mm, # 1.5) and a slide (Menzel Gläser, 76 mm × 26 mm × 1.0 mm) inside a 0.12 mm thick (13 mm chamber diameter) imaging gasket (Grace Bio-Labs, SecureSeal^TM^). The copper grid was orientated such that the 300 nm sections were facing the coverslip.

### Data analysis

The experimental shot noise was evaluated by taking the statistical distribution of the measured FWM field (both in the in-phase and in-quadrature components detected by the lock-in amplifier) in a spatial region where no FWM is present. The standard deviation of this distribution was deduced and was found to be identical in both components, as well as for the co-polarised and cross-circularly polarised components, as expected for an experimental noise dominated by the shot noise in the reference beam^15^. The error bars in the FWM field ratio in Fig. 2 are calculated by propagating the errors from the experimental shot noise in the co- and cross-circularly polarised components, and are shown as two standard deviations. The FWM field ratios in Fig. 2 were measured from the two in-plane data sets 0.5 µm apart in *z* forming the overview in Fig. 1c. Notably, the FWM ratio values are slightly dependent on the axial position of the AuNP. Hence, care was taken to consider the ratio only for NPs that were in focus, based on the maximum co-polarised FWM amplitude detected and on the width of the point-spread function (see SI Fig. S4).

The fitted ellipses to the TEM images in Fig. 2 are obtained using the “Analyse particles - fit ellipse” command in the freely available Java-based image analysis programme ImageJ (https://imagej.net/). This command measures and fits objects in thresholded images. It works by scanning the selection until it finds the edge of an object. It then provides the major and minor semiaxis and the orientation angle *γ* of the best-fitting ellipse. The orientation angle is calculated between the major axis and a line parallel to the *x* axis of the image (see the sketch in Fig. 2). For the ellipses shown by the yellow lines in the TEM images in Fig. 2, the “auto-threshold” default option was applied. To estimate the error bars in the fitted aspect ratios and in the angle *γ*, TEM images were re-fitted using a different threshold such that the area of the fitted ellipse was 80% of the area obtained with auto-threshold, as shown in SI Fig. S3. The horizontal errors bars in Fig. 2 are the single-sided distances between the values using the auto-threshold option and the re-fitted values.

### Centroid fitting

To determine the centroid position of the NPs, we have fitted the spatially resolved FWM field with a Gaussian complex function given by

$$\begin{array}{*{20}{l}} {G\exp \left[ {i{{{\mathrm{{\Phi}}}}} - \frac{{4\log \left( 2 \right)}}{{w^2}}\left( {\epsilon \left( {\left( {x - x_0} \right)\cos {\uptheta}} \right.} \right.} \right.} {\left. { - \left( {y - y_0} \right)\sin {\uptheta}} \right)} \\ \qquad\qquad{ +\, \left( {1/\epsilon } \right)\left( {\left( {x - x_0} \right)\sin {\uptheta}} \right.} {\left. {\left. { - \left( {y -\, y_0} \right)\left. {\cos {\uptheta}} \right)} \right)^2} \right]} \end{array}$$

where *G* is the amplitude of the signal at the peak, Φ its phase, *w* a mean width of the peak, *x*_0_ and *y*_0_ the coordinates of the centroid, ϵ the ellipticity of the peak and *θ* the orientation.

### Affine transformation

We use the linear transformation between the coordinates of image A and the coordinates of image B

2 $${{{\mathbf{r}}}}_{{{\mathbf{B}}}} = {{{\mathbf{C}}}}\left( {{{{\mathbf{r}}}}_{{{\mathbf{A}}}}} \right) = {{{\mathbf{HSRr}}}}_{{{\mathbf{A}}}} + {{{\mathbf{T}}}}$$

with the shear (**H**), scaling (**S**), rotation (**R**) and translation (**T**), given by

3 $$\begin{array}{*{20}{c}}{{{{\mathbf{H}}}} = \left( {\begin{array}{*{20}{c}} 1 & h \\ 0 & 1 \end{array}} \right)}\\ {{{{\mathbf{S}}}} = \left( {\begin{array}{*{20}{c}} {s_x} & 0 \\ 0 & {s_y} \end{array}} \right)}\\ {{{{\mathbf{R}}}} = \left( {\begin{array}{*{20}{c}} {\cos {\upalpha}} & { - \sin {\upalpha}} \\ {\sin {\upalpha}} & {\cos {\upalpha}} \end{array}} \right)}\\ {{{{\mathbf{T}}}} = \left( {\begin{array}{*{20}{c}} 0 & {t_x} \\ 0 & {t_y} \end{array}} \right)}\end{array}$$

where *t*_x_ and *t*_y_ are the component of the translation vector between the two systems, *α* the rotation angle, *s*_x_ and *s*_y_ the scaling factors and *h* the shear between the transformed axes.

To determine the transformation parameters, we identify the same objects (i.e., nanoparticles) in the two images and estimate their coordinates. We then perform a nonlinear least-squares fitting of the parameters, minimising the quantity

4 $$\mathop {\sum}\nolimits_i {\left| {r_{B_i} - C\left( {r_{A_i}} \right)} \right|^2}$$

where *i* counts the objects. Knowing **C**, image A can be transformed into the reference system of image B by transforming the coordinate of each pixel in A and interpolating the corresponding intensity to map the position of the pixels in B.

For the case of nanoparticle clusters in FWM, the centroid coordinate position of each nanoparticle cluster from the FWM z-stack was calculated using the “3D object counter” plugin in ImageJ.

### TEM

Following the FWM analysis, the grids were recovered for TEM analysis by flooding the space between the coverslip and slide with excess water and gently lifting the coverslip^34^. The grid was subsequently dried and transferred to a 120 kV or 200 kV transmission EM (Tecnai12 or Tecnai20 respectively, FEI, now Thermo Scientific). The site of interest was retraced using the outline of the sections and calculating the approximate position of the cell(s) of interest. Overview images were collected, followed by subsequent zooms into the area of interest. No fiducials were added, as they are not required in the reported single AuNP probe CLEM.

### FWM-CLEM workflow

**Fig. 7:**
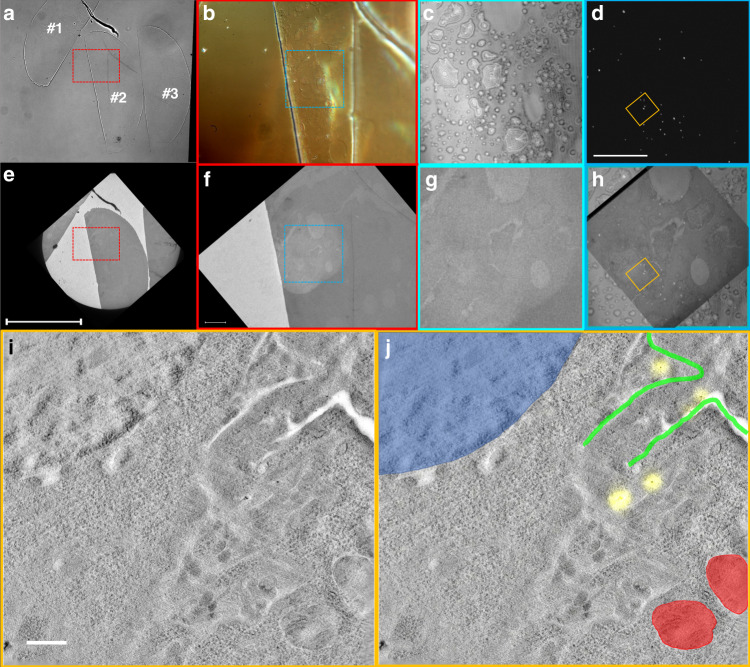
FWM-CLEM workflow. Sections are first visualised by bright-field transmission and DIC microscopy, followed by simultaneous reflection and FWM imaging. Sections are then retrieved for TEM analysis, where the same cell regions are identified. **a** Bright-field transmission microscopy overview image using white-light illumination (halogen tungsten lamp V2-A LL 100 W; Nikon) with a 0.72NA dry condenser, a 10 × 0.3 NA dry objective, and a monochrome CCD camera (Hamamatsu Orca-285). It shows three sections with some folds in section 2 and another imperfection at the top of section 2. **b** Higher magnification DIC image (red boxed area from a), using white-light illumination, a 1.34 NA oil condenser, a 60 × 1.27 NA water objective and a colour consumer camera (Canon EOS 40D); the folds are visible as well as the outlines of cells and other features in the section. **c** Confocal reflection of the corresponding blue boxed area (on an amplitude log scale from 0.1 mV (black) to 200 mV (white) rms detected). **d** FWM acquired simultaneously with reflection, as a maximum amplitude projection over a z-stack (on an amplitude log scale, contrast adjusted for visual purposes). **e** The grid is retrieved for TEM analysis and a similar overview image is acquired. **f** Higher magnification TEM showing the same folds and outlines as in (**b**), as highlighted by the blue frame. **g** Magnified crop of the TEM area in (**f**) where parts of the cells are recognised as seen in confocal reflection c. **h** Overlay of the reflection, FWM and TEM area. FWM was acquired with a pump-probe delay time of 0.5 ps, pump (probe) power at the sample of 30 µW (15 µW), 1-ms-pixel dwell time, pixel size in plane 72 nm, 500 nm step size in *z* and 13 z steps (6 µm total range). A high-resolution TEM image is shown in (**i**) for the region indicated by the orange frame in (**d**) and (**h**). An overlay of the AuNPs seen in FWM (yellow) and this TEM area is provided in (**j**), where the nucleus has been highlighted in blue, the mitochondria in red and segments of the plasma membrane in green. Scale bar in (**e**) is 250 μm and also applies to (**a**); scale bar in (**f**) is 20 μm and also applies to (**b**); scale bar in (**d**) is 20 μm and also applies to (**c**), (**g**) and (**h**); scale bar in (**i**) is 1 μm and also applies to (**j**)

An example of this workflow is described in Fig. 7. As a first step, a bright-field light transmission microscopy overview image is acquired, see Fig. 7a. It shows 3 sections with some folds in section 2 and another imperfection at the top of section 2. Fig. 7b is a higher magnification differential interference contrast (DIC) image (red boxed area from Fig. 7a) where the folds are visible as well as the outlines of cells and other features in the section. Fig. 7c shows the confocal reflection of the corresponding blue boxed area, and Fig. 7d the FWM acquired simultaneously with reflection, as a maximum amplitude projection over a z-stack (on an amplitude log scale, contrast adjusted for visual purposes; see figure caption for details of the acquisition parameters). In Fig. 7e, the grid is retrieved for TEM analysis and a similar overview image is acquired (compare Fig. 7a). Fig. 7f shows the same folds and outlines as in Fig. 7b, as highlighted by the blue frame. Fig. 7g is a magnified crop of the TEM area in Fig. 7f where parts of the cells are recognised as seen in confocal reflection Fig. 7c. Fig. 7h shows an overlay of the reflection, FWM and TEM area. A high-resolution TEM image is shown in Fig. 7i for the region indicated by the orange frame in d,h. An overlay of the AuNPs seen in FWM (yellow) and this TEM area is provided in Fig. 7j, where the nucleus has been highlighted in blue, the mitochondria in red and segments of the plasma membrane in green.

## Supplementary information

Supplementary Information

Supplementary Video SV1

## Data Availability

Information on the data underpinning the results presented here, including how to access them, can be found in the Cardiff University data catalogue at 10.17035/d.2022.0217754089.
