# Supplementary material for: Correlative light-electron microscopy using small gold nanoparticles as single probes
Source: Light Sci Appl. 2023 Mar 29;12:80. doi: 10.1038/s41377-023-01115-4 (PMC10050153; doi:10.1038/s41377-023-01115-4)
Supplement: Supplementary file 1 — Supplementary Information [file 41377_2023_1115_MOESM1_ESM.pdf]

Supplementary Information - Correlative light electron microscopy using small gold nanoparticles as single probes

Iestyn Pope[1], Hugh Tanner[2,4], Francesco Masia[1], Lukas Payne[1], Kenton Paul Arkill[2,5], Judith Mantell[2], Wolfgang Langbein[3], Paola Borri[1*] and Paul Verkade[2*]

[1]School of Biosciences, Cardiff University, Museum Avenue, Cardiff, CF10 3AX, UK.
[2]School of Biochemistry, University of Bristol, University Walk, Bristol, UK.
[3]School of Physics and Astronomy, Cardiff University, The Parade, Cardiff, CF24 3AA, UK.
[4]Present Address: Department of Chemistry, Umeå University, Umeå, 90187, Sweden.
[5]Present Address: School of Medicine, University of Nottingham, Nottingham, NG7 2RD, UK.

*Corresponding author(s). E-mail(s): BorriP@cardiff.ac.uk; P.Verkade@bristol.ac.uk;

S1 FWM phase versus axial position

We have shown in our previous work that the phase Φ^{+}_{FWM} of the co-circularly polarised FWM field encodes the axial displacement between particle and the focus center, thus it can be used to determine the particle z coordinate without axial scanning [1]. This can be easily understood as due to the optical path length difference between the particle and the observation point. For a plane wave of wavevector $k = 2\pi n/\lambda$ with the refractive index n of the medium, the phase would be $2kz$, the factor of 2 accounting for double path in reflection geometry. We have measured Φ^{+}_{FWM} while moving the NP axial position using the sample nano-positioning stage, on a set of 10nm radius AuNPs (see Fig.S1; AuNP 4 to 7 are the same as in Fig. 1c while AuNP 16 is additional). We find a linear relationship with a slope $dz/d\Phi = 34.2$ nm/rad, slightly larger than $\lambda/(4\pi n) = 28.8$ nm/rad. This is due to the propagation of a focussed beam with high NA where a Gouy phase shift occurs, reducing the wavevector in axial direction due to the wavevector spread in lateral direction. The measured slope is consistent with our previous work [1].

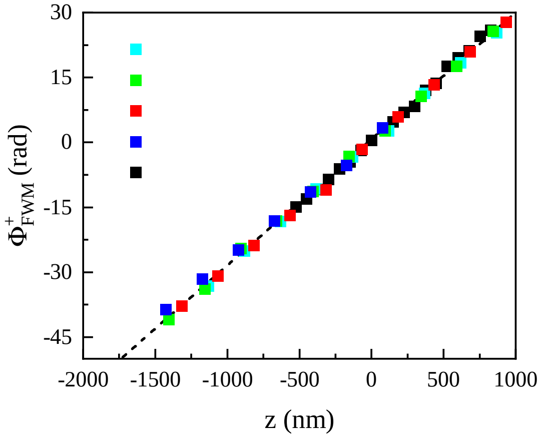

Fig. S1 FWM phase versus NP axial position. Phase Φ^{+}_{FWM} of the co-circularly polarised FWM field measured on a set of individual 10 nm radius AuNPs while scanning their axial position using the sample nano-positioning stage. The phase has been unwrapped by multiples of 2π, and shows a linear dependence on the axial position, as indicated.

S2 Analysis of ellipse shapes in TEM

It was shown in our previous work [1] that using a polarisation-resolved configuration in the FWM field detection provides additional information on the

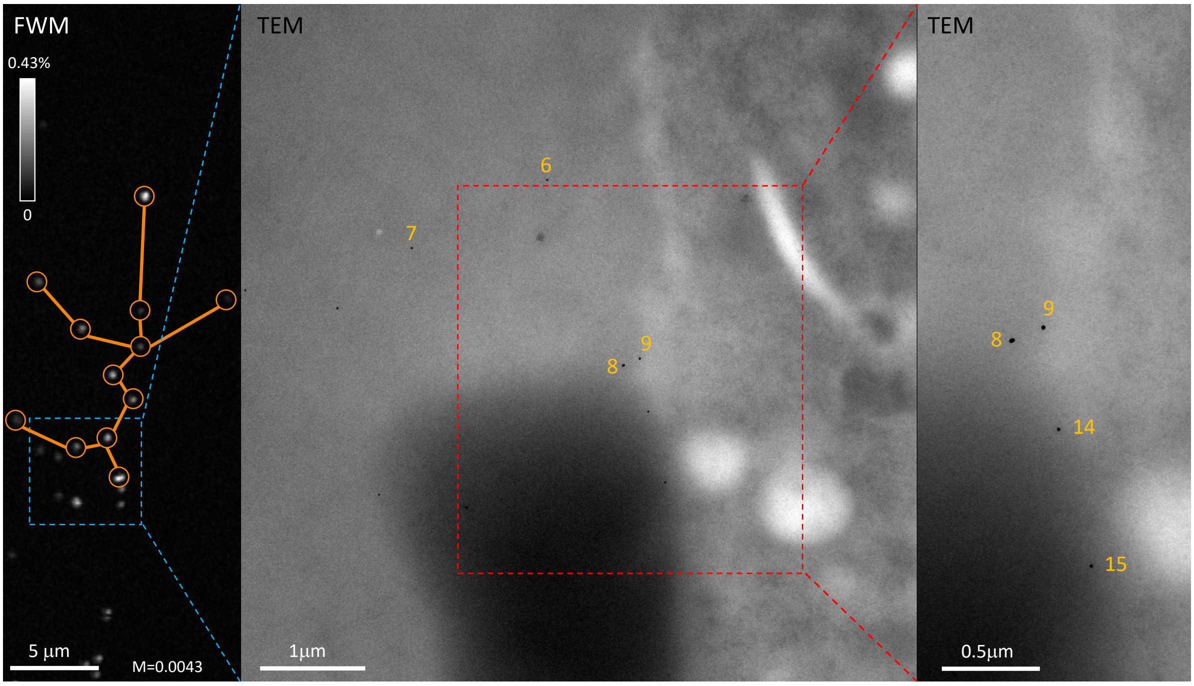

Fig. S2 Additional 10nm radius AuNPs for shape analysis. Overview TEM compared with the FWM imaging in Fig. 1c showing additional AuNPs, numbered as 14 and 15, below those indicated as number 8 and 9. For AuNP 15 a high magnification TEM was acquired and was included in the shape analysis in Fig. 2.

AuNP shape and orientation. Using the CLEM workflow demonstrated here, we have correlatively analysed the measured ratio of the cross to co-circularly polarised FWM field, in amplitude ($A^-_{\rm FWM}/A^+_{\rm FWM}$) and phase ($\Phi^-_{\rm FWM} - \Phi^+_{\rm FWM}$), with the AuNP shape obtained by TEM, and compared the results with the ellipsoid model previously developed [1]. Fig. 2 shows the results on a set of AuNPs as labelled in Fig. 1c plus an additional AuNP (numbered as 15) for which a high-magnification TEM was also acquired. An overview showing the location of this particle below the AuNP pair 8 and 9 is given in Fig. S2.

As discussed in the Methods section, the fitted ellipses to the TEM images in Fig. 2 are obtained using the "Analyse particles - fit ellipse" command in the freely available Java-based image analysis program ImageJ [2]. This command measures and fits objects in thresholded images. It works by scanning the selection until it finds the edge of an object. It then provides the major and minor semi-axis and the orientation angle γ of the best fitting ellipse. The orientation angle is calculated between the major axis and a line parallel to the x-axis of the image. For the ellipses shown by the yellow lines in the TEM images in Fig. 2, the "auto-threshold" default option was applied. To estimate the error bars in the fitted aspect ratios and in the angle γ, TEM images were re-fitted using a different threshold such that the area of the fitted ellipse was 80% of the area obtained with auto-threshold, as shown in Fig. S3. The horizontal errors bars in Fig. 2 are the single-sided distances between the values using the auto-threshold option and the re-fitted values.

S3 FWM Ratio

As also discussed in the Methods section, the FWM field ratios in Fig. 2 were measured from the two in-plane data sets 0.5 µm apart in z forming the overview in Fig. 1c. However, the FWM ratio values are slightly dependent on the axial position of the AuNP. Hence, care has to be taken to consider the ratio only for NPs that are in focus, based on the maximum co-polarised FWM amplitude detected ($A^+_{\rm FWM}$) and on the width of the point-spread function (PSF). An overview of $A^+_{\rm FWM}$, the ratio $A^-_{\rm FWM}/A^+_{\rm FWM}$, and the full-width at half maximum (FWHM) of the $A^+_{\rm FWM}$ profile in plane along the x and y directions is shown in Fig. S4 comparing each NP at the two data sets 0.5 µm apart in z. The filled symbols indicate the values at the plane

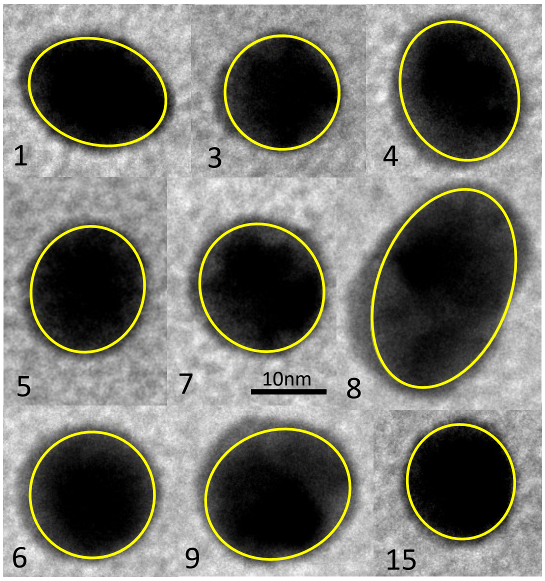

Fig. S3 Fits of ellipse shape on TEM. High-magnification TEM images of selected nanoparticles (numbered as in Fig. 1c with the addition of AuNP 15) fitted with an ellipse shape (shown in yellow) using a threshold such that the area of the fitted ellipse is 80% of the area shown in Fig. 2.

of best focus, used for Fig. 2, while the color is coding each plane, as indicated. For each particle, we see that at the plane of highest amplitude A^{+}_{FWM} the PSF width is small and symmetric in x and y, while in the other plane the PSF width increases and in some cases becomes highly asymmetric (see e.g. AuNP 5) due to aberrations. Note that for AuNP 8 and 9 it was not possible to determine a PSF width along x, because these two AuNPs are too close to each other, hence only the width in y is given. Note also that AuNP 14 was excluded from the analysis in Fig. 2 because its PSF width was found to be significantly asymmetric in both planes.

S3.i Model of FWM ratio versus AuNP ellipticity

To explain the model, we start with a description of the polarizability of the AuNP [3]. In its own reference frame, we choose for the semi-axes (a, b, c) of an ellipsoidal AuNP to be aligned, respectively, along the orthogonal axes (x', y', z') of a Cartesian coordinate system in the positive directions. The polarizability tensor describing the AuNP in its own reference frame is then given by

$$\hat{\alpha}' = \begin{pmatrix} \alpha_a & 0 & 0 \\ 0 & \alpha_b & 0 \\ 0 & 0 & \alpha_c \end{pmatrix}. \tag{S1}$$

The components of $\hat{\alpha}'$, $(\alpha_a, \alpha_b, \alpha_c)$ are related to the unmodified (pump-modified) relative complex permittivity of the AuNP, ε_u (ε_p), and the relative permittivity of the surrounding medium, ε_m, with

$$\alpha_j = V\varepsilon_0 \frac{\varepsilon - \varepsilon_m}{\varepsilon_m + L_j(\varepsilon - \varepsilon_m)}, \tag{S2}$$

where, $V = \frac{4}{3}\pi abc$, is the volume of the AuNP, L_j are the factors describing the geometry of the ellipsoidal shape, ε has the value of either ε_u for a particle in the absence of the pump, or ε_p in presence of the pump pulse at 0.5 ps delay, and ε_0 is the permittivity of free space. We assume ε_m to be constant as a function of the wavelength, λ, of the incident radiation. L_j are found from the analytical expressions,

$$L_j = \frac{abc}{2}\int_0^\infty \frac{dq}{(j^2+q)\sqrt{(a^2+q)(b^2+q)(c^2+q)}} \tag{S3}$$

The AuNP polarizability is transformed into the laboratory reference frame, whose axes we label (x, y, z), with

$$\hat{\alpha} = \hat{R}\hat{\alpha}'\hat{R}^T, \tag{S4}$$

We define $\hat{R}$ and $\hat{R}^T$ as

$$\hat{R} = R_\psi R_\theta R_\phi, \qquad \hat{R}^T = R_\phi^T R_\theta^T R_\psi^T \tag{S5}$$

with

$$R_\phi = \begin{pmatrix} 1 & 0 & 0 \\ 0 & \cos(\phi) & -\sin(\phi) \\ 0 & \sin(\phi) & \cos(\phi) \end{pmatrix}, \tag{S6}$$

$$R_\theta = \begin{pmatrix} \cos(\theta) & 0 & \sin(\theta) \\ 0 & 1 & 0 \\ -\sin(\theta) & 0 & \cos(\theta) \end{pmatrix}, \tag{S7}$$

$$R_\psi = \begin{pmatrix} \cos(\psi) & -\sin(\psi) & 0 \\ \sin(\psi) & \cos(\psi) & 0 \\ 0 & 0 & 1 \end{pmatrix}. \tag{S8}$$

The AuNP permittivity is modelled with the function [4]

$$\varepsilon_u = 1 - \frac{\omega_P^2}{\omega(\omega + i\Gamma)} + \varepsilon^b(\omega) \tag{S9}$$

where $\omega = 2\pi c/\lambda_0$, c is the speed of light in vacuum, Γ is the relaxation rate, ε^b is the contribution

due to bound electrons associated with interband transition from the d bands to the conduction band, and $\omega_P = n_e e^2/\varepsilon_0 m_0$ is the plasma frequency (n_e, e, m_0 are the conduction electron density, charge, and effective mass, respectively).

The permittivity as a function of the optical excitation is modelled as described in Ref.[4]. We call the value without excitation ε_u, and determine the value ε_p with excitation by a pulse at 550 nm wavelength, 0.1pJ/µm^2 fluence and 0.5 ps delay.

The polarization of the AuNP is given by

$$\mathbf{p} = \hat{\alpha}\mathbf{E} \tag{S10}$$

where $\mathbf{E}$ is the incident field and the bold font indicates that it is a vector quantity. The pump field induces a change in the polarizability given by

$$\Delta\hat{\alpha} = \hat{\alpha}_u - \hat{\alpha}_p, \tag{S11}$$

using ε_u and ε_p, respectively. The FWM field resulting from the subsequent probe of the AuNP is proportional to the modified polarizability and the probe field, so that in suited units we can write $\mathbf{E}_{FWM} = \Delta\hat{\alpha}\mathbf{E}_2$, where $\mathbf{E}_2$ is the probe field.

We note that $\mathbf{E}_2$ has circular polarisation at the sample, and choose to compactly represent it here using the Jones vector

$$\mathbf{E}_2 = \begin{pmatrix} 1 \\ i \\ 0 \end{pmatrix}. \tag{S12}$$

We define the co- and cross-polarised components of $\mathbf{E}_{FWM}$ as

$$\mathbf{E}_+ = \mathbf{E}_2^\star \cdot \Delta\hat{\alpha}\mathbf{E}_2 \tag{S13}$$

and

$$\mathbf{E}_- = \mathbf{E}_2 \cdot \Delta\hat{\alpha}\mathbf{E}_2, \tag{S14}$$

respectively, where ($\star$) indicates the complex conjugate.

Hence, we can determine the amplitude and phase ratios of the co- and cross-polarized projections of the FWM field from

$$\frac{A^-_{FWM}}{A^+_{FWM}} = \left|\frac{\mathbf{E}_-}{\mathbf{E}_+}\right| \tag{S15}$$

and

$$\Phi^-_{FWM} - \Phi^+_{FWM} = \arg\left(\frac{\mathbf{E}_-}{\mathbf{E}_+}\right). \tag{S16}$$

For the plot in Fig. 2, when the NP a and c axis are tilted by 45 degrees in the x,z plane, the projected minor to major axis ratio in the x-direction is given by $\sqrt{2}/\sqrt{(a/b)^2+1}$ for the prolate case ($a > b$) and by $\sqrt{(a/b)^2+1}/\sqrt{2}$ for the oblate case ($a < b$).

S4 FWM-EM correlation accuracy

As stated in the paper, we evaluate the correlation accuracy using the coordinates of AuNPs measured in FWM and in TEM, and transforming the coordinates of AuNPs from FWM into the reference system of the TEM image, using a linear transformation. Apart from AuNP 2 which was excluded due to its low FWM amplitude and atypical TEM contrast (see Fig. S5), we applied selection criteria to exclude AuNPs which are too out of focus in FWM by performing the following analysis. For all AuNPs in the dataset, we considered the FWHM of the A^+_{FWM} profile in plane crossing the AuNP centre along the x and y and the error in the centroid localisation from Gaussian fits of the line-profiles. This is shown in Fig. S6 for the 10 nm-radius AuNPs in Fig. 1 and Fig. S7. We excluded AuNPs having a FWHM larger than 0.4 µm as deemed too out of focus, hence subject to localisation uncertainties originating from aberrations in the microscope objective as well as deformations of the pioloform layer supporting the resin section which change from FWM in water to TEM in vacuum. These excluded AuNP 10 and 17, for which the centroid localisation error was found to be around 6 nm, more than twice the average value observed for AuNPs in focus. By inspecting the localisation error, we then also excluded AuNP 13 and 19, which had a centroid localisation error around 10 nm (and a FWHM near the 0.4 µm cut-off), to ensure that the localisation precision is not the limiting factor in the correlation analysis.

Since the resin section is only 300 nm thick, smaller than the axial extension of the PSF in FWM imaging (as shown in Fig. 1b) one would not expect to have AuNPs out of focus. On the other hand, we observed that the pioloform layer supporting the resin section during FWM imaging was not flat but rather exhibited bending and wrinkles. Indeed, by exploiting the topography information encoded in the phase of the reflected probe field, we reconstructed a height profile for the region imaged in Fig. 1 and Fig. S7. This was achieved by unwrapping the phase profile (i.e. removing 2π phase jumps to have a continuous phase change) and using the relationship between phase and

axial position from the slope $dz/d\Phi = 34$ nm/rad (see also Fig.S1). The resulting height profile is shown in Fig. 4 and illustrates that AuNP 10,13,17, and 19 are indeed positioned at a significantly different height than the others, consistent with them appearing out of focus compared to other AuNPs in the image.

Selection criteria applied to the 5 nm-radius AuNPs are shown in Fig. S8. Also here, we considered the FWHM of the A^{+}_{FWM} profile in plane crossing the AuNP centre along the x and y and the error in the centroid localisation from Gaussian fits of the line-profiles. As for the 10 nm-radius AuNP selection criteria, particles having a FWHM larger than 0.4 µm were excluded, which led to excluding AuNP 20. By inspecting the localisation error, we see that for all particles this is larger (at least twice) than what observed for the 10 nm-radius AuNPs in Fig. S6, as expected considering the lower signal to noise ratio from the scaling of the FWM field amplitude with the AuNP size. We therefore applied a higher cut-off to this dataset, and excluded AuNPs having a centroid localisation error larger than 11 nm, to retain the majority of 5 nm-radius AuNPs, while the cut-off is still significantly below, and thus not limiting, the final accuracy found.

An example of CLEM with FWM imaging using 5 nm-radius AuNPs in HeLa cells is shown in Fig. 5. Several AuNPs are clearly visible in both FWM and TEM. A few AuNPs are too close to be spatially distinguished in the FWM image, but 19 individual AuNPs are available for position analysis. This resulted in a correlation accuracy of 58 nm, retaining 13 AuNPs for the correlation (see orange circles in Fig. 5), while 6 AuNPs were excluded (white circles in Fig.5) based on the criteria discussed above. Another example showing an adjacent region is provided in Fig. S9 where again 13 individual AuNPs were used for the correlation. Merging both regions results in a correlation accuracy of 63 nm.

Considering that the shot-noise limited precision in locating the centroid position of a AuNP in focus by FWM is only a few nanometres, the measured correlation accuracy is limited by systematic errors. To address this point, we performed FWM-CLEM using 10 nm-radius AuNPs whereby the coordinates of the particles in FWM were measured in 3D with a fine axial scan (50 nm step size in z), such that the coordinates at the plane of optimum focus are accurately determined and systematics from e.g. out-of-focus aberrations are eliminated. These results are summarized in Fig. 6. AuNPs form small clusters and are no longer resolved as individual particles in FWM. Therefore, in this case, we determined the centroid position of the cluster in 3D from the FWM z-stack (see Methods), and compared its 2D in-plane coordinates with the position of the geometrical centre of the cluster in TEM (which is a 2D transmission projection) for the correlation analysis. The resulting correlation accuracy for the six clusters shown in Fig. 6 is 36 nm. Another example correlating 10 clusters is provided in Fig. S10, for which an accuracy of 44 nm is found.

We highlight that the analysis of the correlation accuracy shown here considers AuNPs as useful identifiable objects visible in both EM and FWM, with no need for additional fiducial markers. Hence, besides the exclusion criteria discussed above, all AuNPs are used to calculate the linear transformation matrix **C** that minimises the deviations in the coordinate overlay, as explained in the main article. For comparison, we have examined the case of choosing three AuNPs as fiducials to determine the matrix transformation, and calculated the quantity S for the remaining particles. We have used the AuNPs shown in Fig. 1c, and considered different choices of AuNPs fiducials. The corresponding value S is found to depend significantly on the position of the fiducials, with the smallest $S =$ 78 nm obtained when using AuNPs 1,6,12. If fiducials are close to each other (e.g. AuNP 3,4,5) or positioned mostly along one direction (e.g. AuNP 1,3,7) the overlay error becomes significantly larger with S values of 150-200 nm. Generally, this approach carries a larger error due to the subjective choice of the fiducials, and the propagation in the uncertainty of finding the transformation **C** with only three particles together with the position error of the remaining particles. Since they are visible in both images, using all available AuNPs for the FWM-EM overlay offers a more accurate correlative approach.

S5 Cellular ultrastructure via sampling at different depths in EM

Without metal stains, the EM contrast in the ultrastructural definition of cell organelles is low. Notably, this can be improved using electron tomography and applying an average intensity z-projection onto a subset of reconstructed slices. Fig. S11 shows an example where an electron tomography tilt series has been acquired. Fig. S11a and b are zoomed in areas of

Fig.7i. Following reconstruction of the electron tomogram (see Supplementary Video SV1), a subset of slices was z-projected and the average intensity was acquired using Fiji [5]. Fig. S11a shows the projection of slices 25-35 which highlights 2 endocytic profiles containing gold (red highlighted areas). Fig. S11b shows the projection of slices 45-55 and highlights another endosome containing a gold particle (red highlighted area) which was not visible in a, and an endocytic pit (arrow).

Supplementary Video SV1. Electron tomography. A tilt series of the section from -55 to +55 degrees and 1 degree increments was acquired on a 200kV transmission electron microscope (Tecnai20, LaB6, FEI / Thermo Fisher Scientific) using a Fischione 2040 dual axis tomography holder (dual axis not acquired). The tilt series was reconstructed using the IMOD software package [6] and the internalised gold particles as markers for alignment.

References

[1] Zoriniants, G., Masia, F., Giannakopoulou, N., Langbein, W. & Borri, P. Background-free 3D nanometric localization and sub-nm asymmetry detection of single plasmonic nanoparticles by four-wave mixing interferometry with optical vortices. *Phys. Rev. X* **7**, 041022 (2017).

[2] URL https://imagej.net/.

[3] Payne, L. M., Albrecht, W., Langbein, W. & Borri, P. The optical nanosizer – quantitative size and shape analysis of individual nanoparticles by high-throughput widefield extinction microscopy. *Nanoscale* **12**, 16215–16228 (2020).

[4] Masia, F., Langbein, W. & Borri, P. Measurement of the dynamics of plasmons inside individual gold nanoparticles using a femtosecond phase-resolved microscope. *Phys. Rev. B* **85**, 235403 (2012).

[5] Schindelin, J. *et al.* Fiji: an open-source platform for biological-image analysis. *Nature Methods* **9**, 676–682 (2012).

[6] Kremer, J. R., Mastronarde, D. N. & McIntosh, J. R. Computer visualization of three-dimensional image data using IMOD. *J Struct Biol.* **116**, 71–76.

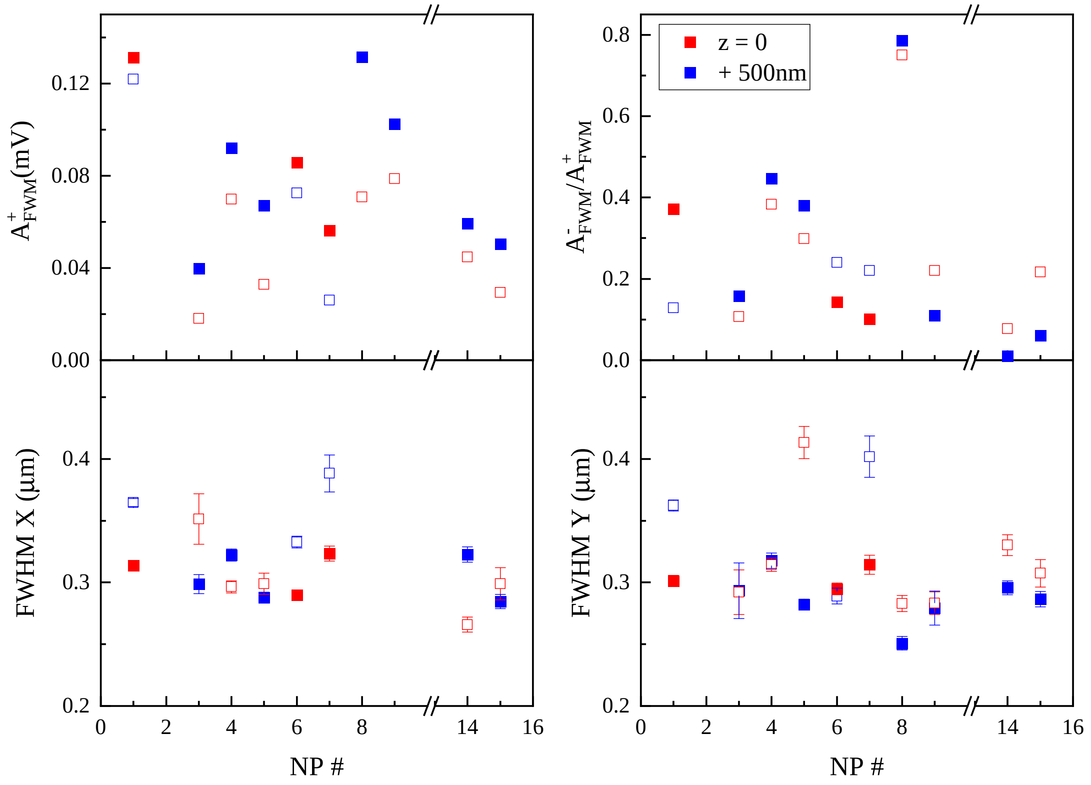

Fig. S4 FWM amplitudes and PSF widths at two focal planes. Overview of A^+_{FWM}, the ratio A^-_{FWM}/A^+_{FWM}, and the full-width at half maximum (FWHM) of the A^+_{FWM} profile in plane along the x and y directions versus NP number, for the AuNPs shown in Fig. 2. Filled (empty) symbols indicate the values at the plane in (out of) focus. The color is coding each plane, as indicated.

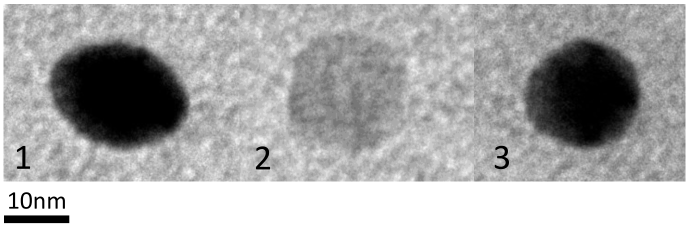

Fig. S5 TEM of AuNP n.2. High magnification TEM showing AuNP 2 compared with 1 and 3.

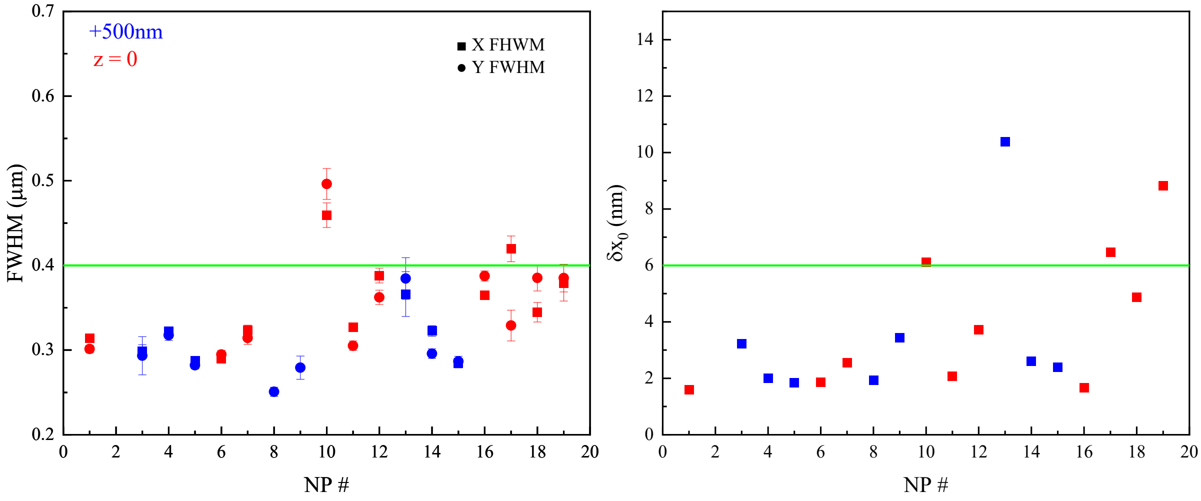

Fig. S6 10-nm radius AuNP selection for CLEM Left: Overview of the FWHM of the A^{+}_{FWM} profile in plane crossing the AuNP centre along the *x* and *y* directions versus NP number, for the AuNPs shown in Fig. 1 and Fig. S7. Right: Error in the centroid localisation from Gaussian fits of A^{+}_{FWM} line-profiles crossing the AuNP centre along the *x* direction. Values are shown at the plane of highest A^{+}_{FWM} amplitude, and the colour is coding each plane, as indicated. Lines show the cut-off criteria for AuNP selection (see text), which resulted in excluding AuNP 10, 13, 17, 19.

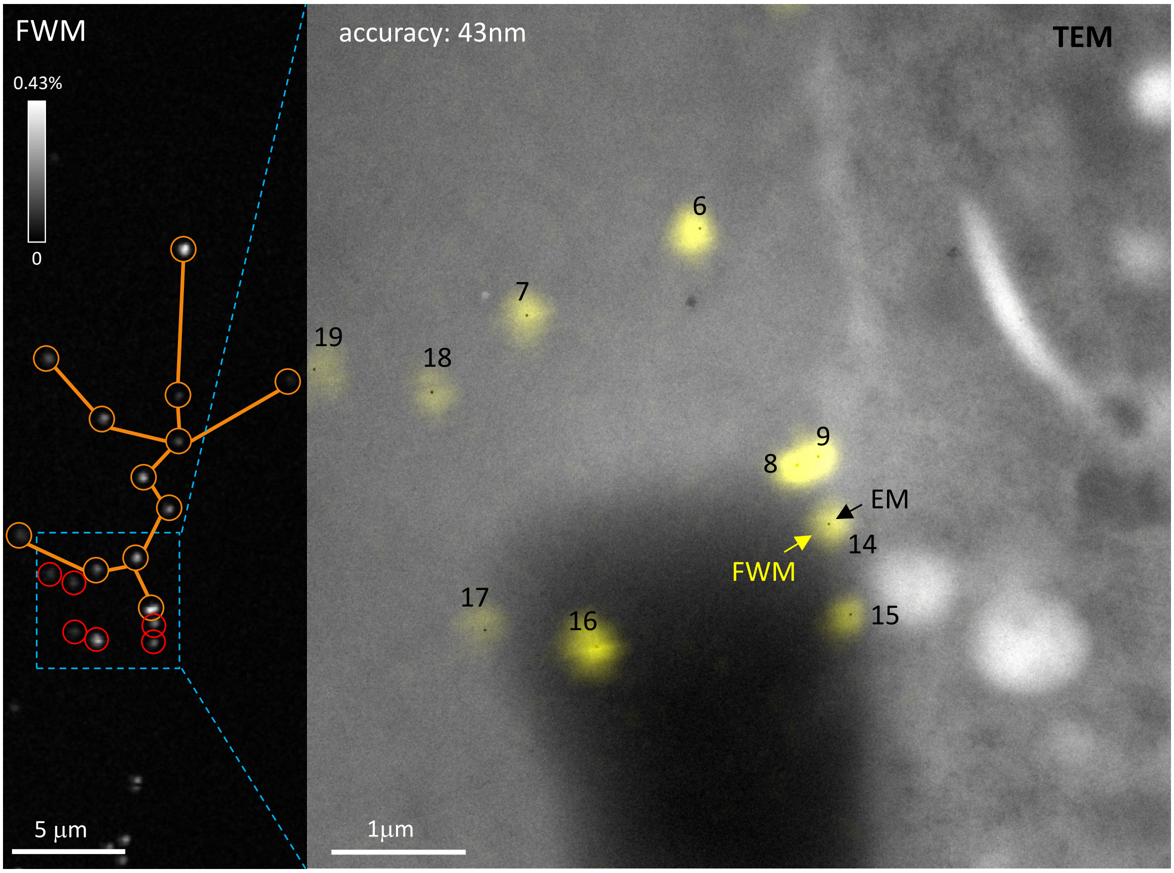

Fig. S7 CLEM correlation accuracy. Overlay of FWM field amplitude and TEM image using a different region compared to Fig. 1c as indicated by the blue frame on the left side (red circles highlight the additional AuNPs). The FWM image is transformed into the EM reference system using a linear transformation matrix that accounts for translation, rotation, shear and scaling of axes. On the right side, yellow spots show individual AuNPs in FWM overlaid onto the EM (black dots). The correlation accuracy is indicated. AuNPs 17 and 19 were out of focus and excluded from the correlation analysis (see text).

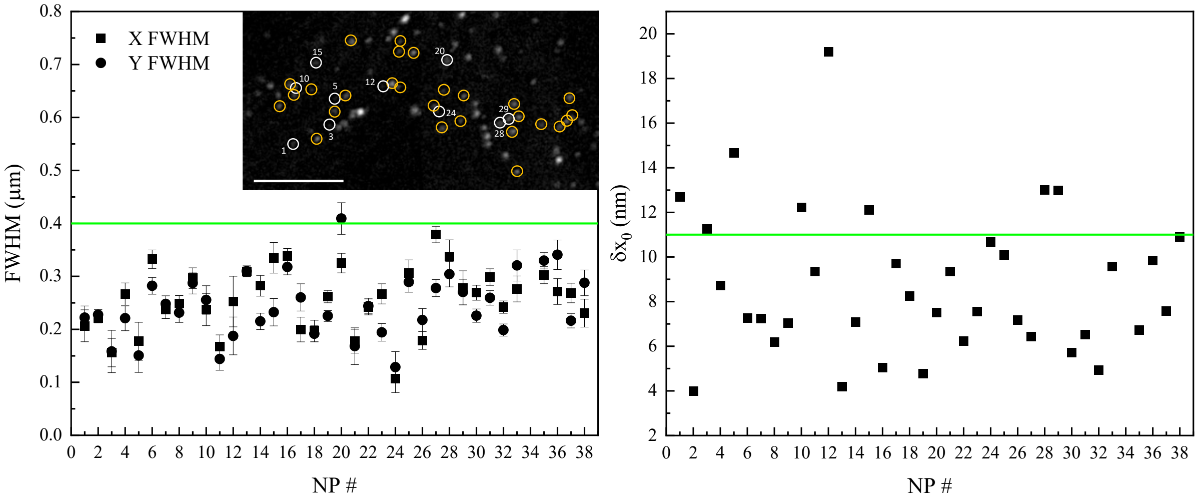

Fig. S8 5-nm radius AuNP selection for CLEM Left: Overview of the FWHM of the A^{+}_{FWM} profile in plane crossing the AuNP centre along the x and y directions versus NP number, for the AuNPs shown in Fig. 5 and Fig. S9. Right: Error in the centroid localisation from Gaussian fits of A^{+}_{FWM} line-profiles crossing the AuNP centre along the x direction. Lines show the cut-off criteria for AuNP selection (see text), which resulted in excluding 6 AuNPs (number 1, 3, 5, 10, 12, 15) for the data shown in Fig. 5 and 4 AuNPs (number 20, 24, 28, 29) for the data in Fig. S9. The inset highlights the excluded AuNPs in the FWM A^{+}_{FWM} image as white circles (with corresponding numbers) and the included AuNPs as yellow circles. Scale bar in the inset image is 5 μm.

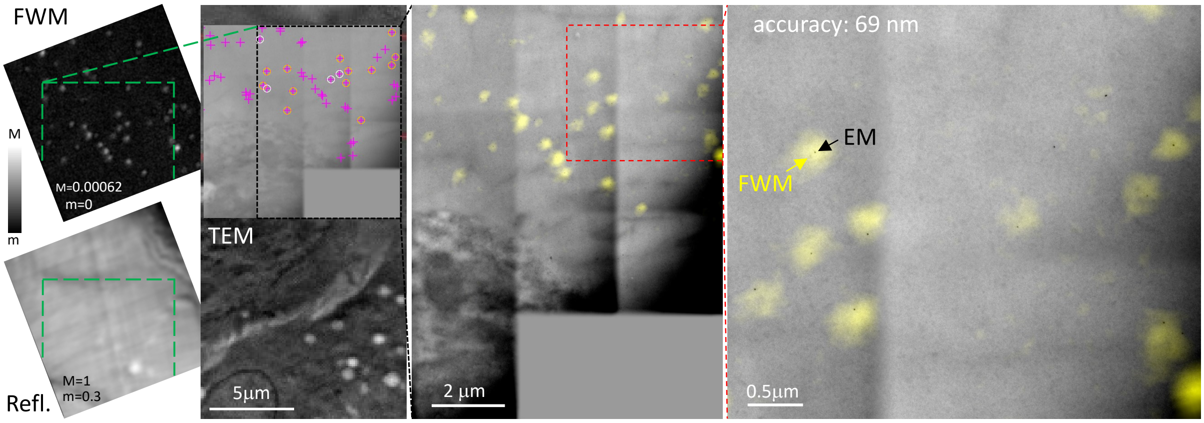

Fig. S9 CLEM correlation accuracy with 5 nm-radius AuNPs. HeLa cells incubated with 5nm-radius AuNPs bound to the EGF protein. CLEM overview on a region adjacent to the one shown in Fig. 5. Individual AuNPs are detected background-free in FWM (left). The confocal reflection image simultaneously acquired with FWM is shown below (linear grey scales are from m to M as indicated; M=1 corresponds to 65 mV rms detected, see Methods for details of the excitation and detection conditions). A large overview TEM of the same region is shown together with a series of high resolution EM images stitched together. Individual AuNPs are highlighted by the circles (see dashed green frame for the corresponding AuNPs in FWM). The overlay between FWM (yellow) and TEM (grey) is shown on the center and further zoomed into the indicated red dashed area on the right side (contrast adjusted to aid visualisation). For the correlation analysis, of the 17 individual AuNPs highlighted by the circles, 4 (white circles) were discarded as being of focus. The FWM image was transformed into the EM reference system using a linear transformation matrix that accounts for translation, rotation, shear and scaling of axes. On the right side, individual AuNPs identified in FWM (yellow spots) are seen in EM (black dots). The correlation accuracy is indicated.

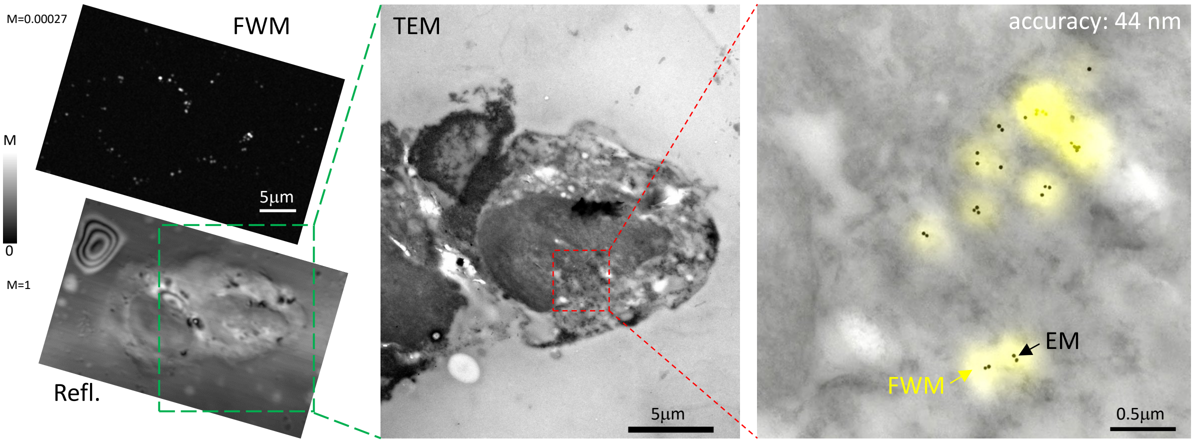

Fig. S10 CLEM correlation accuracy with 3D FWM analysis. FWM-CLEM using 10nm-radius AuNPs bound to EGF internalised in HeLa cells whereby the coordinates of the particles in FWM are measured in 3D via a z-stack. A large FWM overview in 2D with corresponding reflection image measured simultaneously is shown on the left (linear grey scales are from 0 to M as indicated; M=1 corresponds to 33mV rms detected; see Methods for details of the excitation and detection conditions). A TEM overview of the same region is shown in the center, as indicated by the green dashed frame. On the left, an overlay of FWM field amplitude (yellow) and TEM image (grey) is shown for the region highlighted by the red dashed frame, where FWM is a maximum amplitude projection from a 3D z-stack (50 nm step size in *z*). AuNPs form small clusters and are no longer resolved as individual particles in FWM. The centroid position of each cluster was determined in 3D from the FWM z-stack (see Methods), and its 2D in-plane coordinates were compared with the position of the geometrical centre of the cluster in TEM (which is a 2D transmission projection) for the correlation analysis. The resulting correlation accuracy from the comparison of the ten clusters shown in the figure is indicated.

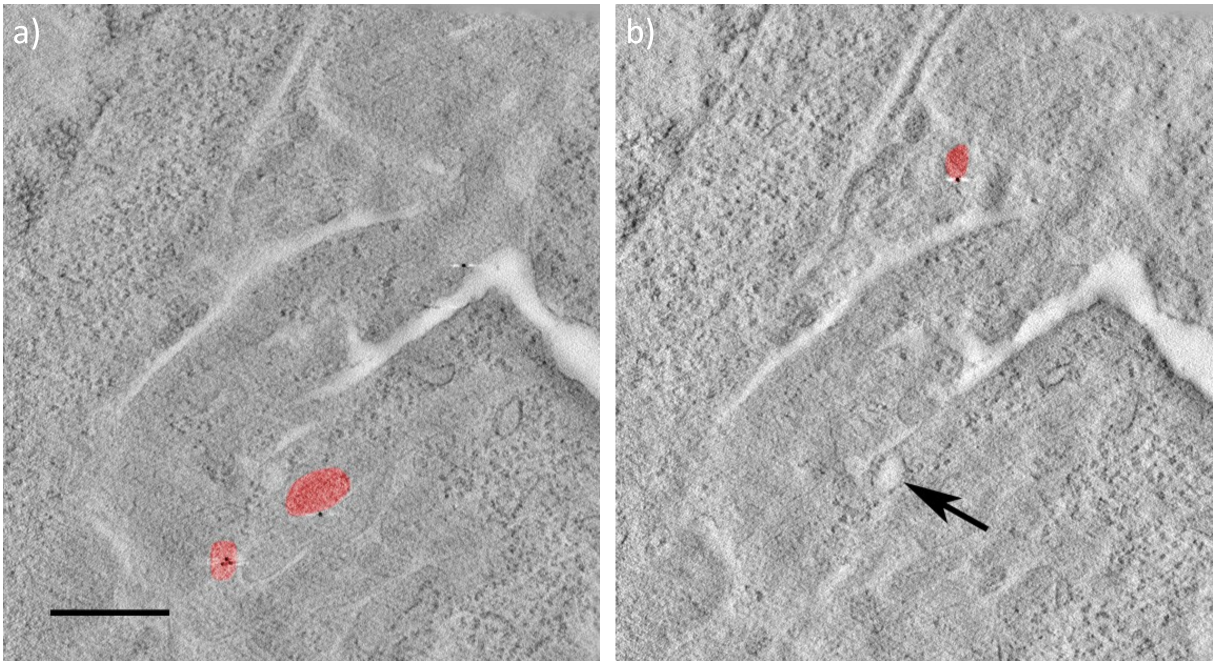

Fig. S11 Visualisation of cellular ultrastructures via sampling at different depths in EM. Following reconstruction of the electron tomogram shown in the Supplementary Video SV1, a subset of slices was z-projected and the average intensity was acquired using Fiji [5]. a) Projection of slices 25-35 which highlights 2 endocytic profiles containing gold (red highlighted areas). b) Projection of slices 45-55 highlighting another endosome containing a gold particle (red highlighted area) which was not visible in a, and an endocytic pit (arrow). Note that a and b show zoomed areas of Fig.7i. Scale bar is 1 µm.
